# Assessing the Impact of Organ Failure and Metastases on Quality of Life in Breast Cancer Patients: A Prospective Study Based on Utilizing EORTC QLQ-C30 and EORTC QLQ-BR45 Questionnaires in Romania

**DOI:** 10.3390/jpm14020214

**Published:** 2024-02-16

**Authors:** Andreea-Iuliana Ionescu (Miron), Alexandra-Valentina Anghel, Ionuț-Lucian Antone-Iordache, Dimitrie-Ionuț Atasiei, Cătălin-Alexandru Anghel, Andrei-Alexandru Barnonschi, Alexandra-Maria Bobolocu, Catinca Verga, Florica Șandru, Horia-Dan Lișcu

**Affiliations:** 1 Department of Oncological Radiotherapy and Medical Imaging, “Carol Davila” University of Medicine and Pharmacy, 020021 Bucharest, Romaniaantoneiordachelucian@stud.umfcd.ro (I.-L.A.-I.); ionut.atasiei@stud.umfcd.ro (D.-I.A.); catalin.anghel@stud.umfcd.ro (C.-A.A.); andrei.barnonschi@stud.umfcd.ro (A.-A.B.); alexandra.c.bobolocu@stud.umfcd.ro (A.-M.B.); catinca.n.verga@stud.umfcd.ro (C.V.); horia-dan.liscu@drd.umfcd.ro (H.-D.L.); 2 Department of Medical Oncology, Colțea Clinical Hospital, 030167 Bucharest, Romania; 3 Department of Dermatovenerology, “Carol Davila” University of Medicine and Pharmacy, 020021 Bucharest, Romania; florica.sandru@umfcd.ro; 4 Department of Dermatology, Elias University Emergency Hospital, 011461 Bucharest, Romania

**Keywords:** breast cancer patients, metastatic breast cancer patients, organ failures, EORTC QLQ-BR45, EORTC QLQ-C30, survival rate, quality of life, QoL

## Abstract

Breast cancer (BC) significantly impacts the quality of life (QoL) of affected individuals. This study, conducted at Colțea Clinical Hospital, Bucharest, aimed to assess the impact of organ failures and metastases on QoL in breast cancer patients using EORTC QLQ-C30 and EORTC QLQ-BR45 questionnaires and the survival rate to understand the clinical journey and the quality of life status in breast cancer patients. From January 2019 to October 2022, a prospective, observational study surveyed 874 patients, revealing 201 fatalities, 66 refusals, and 607 eligible participants. Results indicated statistically significant differences in various QoL aspects for patients experiencing heart failure, including physical functioning, pain, insomnia, global health status, and overall summary score. Kidney failure exhibited significance in physical functioning for QLQ-C30 and body image, sexual functioning, and endocrine sexual symptoms for QLQ-BR45. Respiratory failure demonstrated significant differences across multiple QoL domains. Patients with bone metastases reported lower physical functioning (*p* = 0.006) and increased pain (*p* = 0.002). This study has revealed an overall 5-year life expectancy of 68.8%, with survival rates of 93.8% for Stage I, 86.3% for Stage II, and 77.2% for Stage III breast cancer. Metastatic cancer patients have shown a 35.6% survival rate over 45 months, with a median survival duration of 36 months. A significant limitation of our study was the administration of the questionnaire only once, preventing us from quantifying the impact of specific treatment types on quality of life. This study emphasizes the necessity of using standardized QoL assessments in clinical practice from the initial presentation to ongoing follow-up.

## 1. Introduction

Breast cancer (BC) is a common condition that has a significant impact on the quality of life of patients. According to GLOBOCAN, it is the most prevalent type of malignancy in women around the globe. In 2020, it has been estimated that 2.3 million newly diagnosed cases of breast cancer, being the fifth most common cause of cancer death globally, leading to 685,000 deaths [1]. Investigating breast cancer is a continuing concern within the public health system, especially in low–middle-income countries. Lack of disease awareness, insufficient screening programs, late diagnosis, and a reduced number of medical institutions has led to an increase in mortality and morbidity in breast cancer patients [2,3,4]. Paradoxically, while breast cancer is the most prevalent cancer in the female population, these patients have the highest percentage of survivorship. Elevated rates of curative treatment have been observed for localized disease and long-term survival [5].

Despite the high percentage of survivorship, little progress has been made in assessing the quality of life (QoL) in this specific population. The concept of QoL is defined by the World Health Organization (WHO) as the perception of health status according to their own personal and cultural beliefs and in association with their objectives, expectations, concerns, and standards [6]. Breast cancer diagnosis has a negative impact on QoL, interfering with physical, social, and psychoemotional function [7]. Cella et al. define QoL as being the “gap between one’s actual functional level and one’s ideal standard” [8]. Shumaker et al. proposed the definition “individuals’ overall satisfaction with life and their general sense of personal well-being” [9,10,11]. Scholars have long debated to establish a universal definition regarding QoL. Therefore, to evaluate the QoL as accurately as possible, several questionnaires have been developed. In the context of breast cancer, questionnaires such as the European Organization for Research and Treatment of Cancer Quality of Life Questionnaire Core 30 (EORTC QLQ-C30), European Organization for Research and Treatment of Cancer Quality of Life Questionnaire—Breast Cancer Module (EORTCQLQ-BR45), Functional Assessment of Cancer Therapy—Breast (FACT-B), and Edmonton Symptom Assessment System (ESAS) have been validated and applied in clinical settings [12,13,14,15]. QoL can be influenced, on the one hand, by the direct involvement of malignancy and, on the other hand, by secondary therapies such as mastectomy, systemic therapies, radiotherapy, and hormonal therapy [16,17]. While novel therapies such as hormonal therapy, immunotherapy, and molecular-targeted therapy have improved the survival rate, approaches to enhance the QoL have not been developed accordingly [11,18,19]. Therefore, we consider that this particular group of patients should benefit from research not only on disease-free survival (DFS), overall survival (OS), and progression-free survival (PFS) but on QoL as well.

Currently, QoL is considered a critical final point in clinical studies. It has been concluded that the assessment of QoL in oncologic patients could improve the treatment and serve as a prognostic factor, among other variables. For example, mastectomy, especially bilateral, has been associated with poor QoL [20]. In the case of patients with multiple organ failure, evaluating the QoL could represent a pillar in symptom management due to organ dysfunction, therefore improving the well-being status [21]. Additionally, oncologic patients with a low life expectancy could benefit from the assessment of QoL by helping the oncologist revise the therapeutic scheme.

There are two primary aims of this study: (1). To evaluate the impact of organ failures and metastases upon QoL in breast cancer patients using EORTC QLQ-C30 and EORTC QLQ-BR45 questionnaires; (2) to assess the survival rate in order to understand the clinical journey and the quality of life status in breast cancer patients.

## 2. Materials and Methods

Existing research recognizes the critical role played by the QoL in breast cancer patients. During the Breast Cancer session at European Society for Medical Oncology (ESMO) 2018, Nagi S. El Saghir mentioned that oncologists should carefully monitor patients’ QoL [22]. Measuring the QoL scale should become a primary objective regarding novel therapies due to the fact that some therapeutic agents could prolong survival by only a few months. Moreover, a new term has been proposed—patient-reported outcome (PRO)—to emphasize the importance of active involvement of the patient regarding treatment-related toxicity. PRO is evaluated using tools such as patient-reported outcome measurement (PROM) and aims to determine the patient’s health status [23].

We have conducted a prospective observational study through a survey administered to patients admitted to the Oncology Department of the Colțea Clinical Hospital, Bucharest. This study included all breast cancer patients registered in the Oncology Department from 1 January 2019 to 1 October 2022, regardless of age, stage at diagnosis, molecular subtype, or type of treatment received.

The inclusion criteria were as follows:

- Female patients who were treated solely in the Colțea Clinical Hospital;
- Patients aged over 18 years diagnosed with stage 0–IV breast cancer by histopathological examination.

The exclusion criteria were as follows:

- Male patients;
- Patients who did not give their consent to participate in this study;
- Patients in visceral crisis;
- Patients diagnosed with premalignant breast cancer lesions;
- Patients who became deceased during data collection;
- Patients who presented other types of malignancy.

EORTC QLQ-C30 and EORTC QLQ-BR45 have been used in our study to evaluate QoL in breast cancer patients. The EORTC QLQ-C30 questionnaire includes a set of 30 questions that can be applied to all oncologic patients. It aims to evaluate QoL by analyzing physical, psychological, and social status. It has three main components: functional scales, symptom scales, and global health status. The functional scales address five parameters: physical functioning (PF) (5 questions), role functioning (RF) (2 questions), emotional functioning (EF) (2 questions), cognitive functioning (CF) (2 questions), and social functioning (SF) (2 questions). The symptom burden is determined by several symptoms, such as fatigue (FA), pain (PA), nausea and vomiting (NV), appetite loss (AL), constipation (CO), diarrhea (DI), and financial issues (FI). The EORTC QLQ-BR45 questionnaire comprises a set of 45 questions designed for patients diagnosed with breast cancer. Its purpose is to evaluate both symptomatology and functional aspects through items specifically correlated with breast cancer. The functional scales within the questionnaire gather data concerning satisfaction with body image (BI) (4 questions), breast satisfaction (BS) (2 questions), sexual function (SF) (2 questions), sexual enjoyment (SE) (1 question), and future perspectives (FP) (1 question). Symptomatology assessed by the QLQ-BR45 pertains to systemic therapy side effects, concerns related to hair loss (HU), and symptoms associated with the arm (ARM) and breast (BR) (such as pain or swelling). Additionally, it includes a target therapy symptom scale, encompassing endocrine therapy-related symptoms (ET), cutaneous and mucosal symptoms (SM), and endocrine sexual symptoms (ES). Scoring was made using the EORTC QLQ-C30 and EORTC QLQ-BR45 scoring manuals; both questionnaires have similar principles. Each question has answers ranging from 1 (not at all) to 4 (very much). Global health status was evaluated using two questions (overall quality of life and the patient’s health status), with ratings falling within the range of 1 (very poor) and 7 (excellent). The range is defined as the difference between the possible lowest and highest responses for each item. Thus, for functional and symptom scales, the range is set at 3, while for global health status, the range is 6.

Initially, a raw score is calculated, representing the mean of the answers at the items that compose the corresponding scale.

$$Raw\;Score\left(RS\right)=\frac{I_{1}+I_{2}+\ldots +I_{n}}{n},$$

where n stands for the number of questions that form the scale. Therefore, this RS is converted through a linear transformation in order to obtain an S score with a range of 0–100. Depending on the type of scale, we have applied the following formulas:

For functional scale,

S={1−(RS−1)/range)}×100,where range=3.

For symptom scale,

S={(RS−1)/range}×100,where range=3.

Supplementary data, for QLQ-C30, were calculated using the following scores:

For global health status/QoL,

S={(RS−1)/range}×100,where range=6.

For QLQ-C30 summary score,

S=(PF2+RF2+EF+CF+SF+100−FA+100−PA+100−NV+100−DY+100−SL+100−AP+100−CO+100−DI)/13,

where PF2 denotes physical functioning, RF2 denotes role functioning, EF denotes emotional functioning, CF denotes cognitive functioning, SF denotes social functioning, FA denotes fatigue, PA denotes pain, DY denotes dyspnea, SL denotes insomnia, AP denotes appetite loss, CO denotes constipation, and DI denotes diarrhea.

The obtained scores were analyzed according to the manual’s indications. A higher score for functional scales means better functioning. We considered that scoring <33.33% indicates a significant impairment of QoL, and scoring >66.66% suggests a minor influence on QoL. A higher score on symptom scales implies worse functioning. We considered that scoring <33.33% represents a lower symptom burden, and scoring >66.66% indicates a significant symptom burden given by breast cancer.

In addition to the two questionnaires administered, we have studied the following parameters: the patient’s age at the time of questionnaire administration, performance status (ECOG), menopausal status, body mass index (BMI), the stage at the time of diagnosis, and the type of surgery performed (mastectomy/breast conservative surgery). For patients with metastatic breast cancer, we considered the localization of metastases (osseous and non-osseous). These patients were separated into two groups so we could comparatively assess their quality of life. We compared the quality of life in patients with bone metastases (M1oss) only versus patients with non-osseous metastases (visceral and non-visceral without M1oss). On the other hand, based on the multidisciplinary approach and blood tests, we noted the presence or absence of organ failures: heart failure, respiratory failure, kidney failure, neurologic dysfunction, hepatic failure, and gastrointestinal dysfunction. Therefore, we correlated the results of the quality of life questionnaires with each organ failure.

Heart failure (HF) represents a clinical syndrome caused by the heart’s inability to provide sufficient blood flow for the body’s metabolic needs or fulfill this function at the cost of increasing ventricular filling pressures. In our study, this diagnosis was established by the cardiologist based on medical history, clinical examination, laboratory tests, and echocardiography measuring the left ventricular ejection fraction. Symptoms such as dyspnea or fatigue are common in patients with HF, but they can also be encountered in breast cancer patients without HF.

Renal failure (KF) is a frequently encountered comorbidity among patients diagnosed with breast cancer. In our study, patients with breast cancer who met Kidney Disease Improving Global Outcomes (KDIGO) criteria for acute kidney injury, as well as those diagnosed with chronic kidney disease (characterized by renal dysfunction for at least 3 months), were considered to have renal failure.

Neurological dysfunction (N) is characterized by the impairment of mental status (evaluated by the Glasgow Coma Scale) and motor function. Patients diagnosed with breast cancer may exhibit central nervous system impairment due to brain or spinal cord metastases. Depending on the site of metastasis, clinical manifestations can vary. The most frequent metastatic site of osseous metastasis is the thoracic spine, where symptoms can range from paresthesia to paralysis, respiratory failure, and autonomic nervous system dysfunction through medullary compression. Brain metastases are often diagnosed simultaneously with spinal metastases. Symptoms may be absent or, if present in eloquent areas, can cause specific symptoms or general manifestations such as increased intracranial pressure, nausea, vomiting, and confusion. In our study, we included patients who had a history of neurological dysfunction, such as episodes of loss of consciousness, cerebrovascular accidents, cerebral metastases, or peripheral neuropathy.

Liver failure (LF) is characterized by impaired hepatocyte synthesis function associated with altered mental status. In our study, both patients who presented acute liver injury and those with chronic liver impairment were included. Patients were evaluated using blood tests, especially serum transaminase levels, direct bilirubin, total proteins, serum albumin, alkaline phosphatase, and gamma-glutamyl transferase (GGT) levels. Coagulation times were also assessed.

Respiratory failure (RF) is defined as the inability of the respiratory system to efficiently exchange gases, with hypoxemia +/− hypercapnia being characteristic of this syndrome. In this study, we evaluated the quality of life of breast cancer patients diagnosed during this study with acute respiratory failure, as well as those diagnosed with a form of chronic respiratory failure (chronic obstructive pulmonary disease, asthma, and pulmonary fibrosis).

Gastrointestinal dysfunction (GI) in patients diagnosed with breast cancer involves the development of chemotherapy-associated dyspepsia syndrome, manifested by nausea, vomiting, anorexia, and early satiety. Additionally, patients with digestive toxicity, such as diarrhea, constipation, and mucositis, were included in this category.

**Figure 1:**
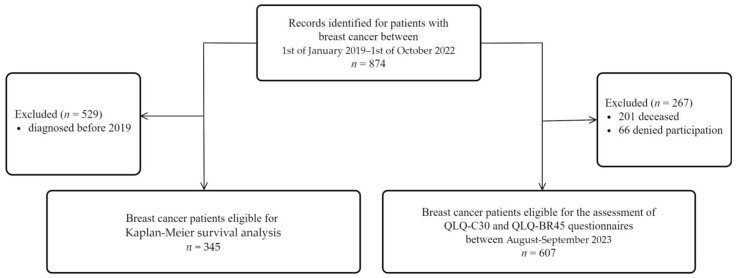
Methodological scheme regarding the eligibility of breast cancer patients included in our study.

Schematic presentation of our study design can be seen in Figure 1.

Between 1 January 2019 and 1 October 2022, 874 breast cancer patients were admitted to the Oncology Department of Colțea Clinical Hospital of Bucharest. The questionnaires were completed in August–September 2023. At the time of the questionnaire administration, of the 874 patients, 201 of them had passed away, 66 did not agree to participate in this study, and 607 patients were eligible for this study. To execute the Kaplan–Meier survival curve, in January 2024, we checked the deceased status, and from September 2023 to January 2024, 10 more patients had passed away. Therefore, between 1 January 2019 and 1 January 2024, 211 deaths were registered for the 874 patients. The Kaplan–Meier survival analysis was conducted exclusively on a cohort of 345 patients who received their diagnosis within the timeframe extending from 1 January 2019 to 1 October 2022.

The questionnaires utilized were supplied by EORTC in their Romanian-translated and validated version. Furthermore, they were completed in Romanian, with the assistance of a medical student participating in this study to address any clarification needed.

### 2.1. Ethical Considerations

This study was approved by the Ethics Committee of Colțea Clinical Hospital, Bucharest, where it took place, following decision No. 19092/5 October 2021.

We obtained consent from the European Organization for Research and Treatment of Cancer (EORTC) to use two quality of life (QoL) questionnaires, receiving them directly translated into Romanian. It should be noted that the EORTC QLQ-C30 is an accepted and validated questionnaire, while the EORTC-BR45 is in the process of validation.

Informed consent was obtained from all patients who participated in this study and completed the questionnaires. Patients were informed about this study’s purpose and assured they would not be subjected to any risks. Additionally, it was explained to them that participation in this study could provide a better understanding of the impact of diagnosis and treatment on their quality of life. This awareness might contribute to more effective management of emotions and disease-related distress. Furthermore, they were informed that this study could help to identify factors influencing the quality of life in breast cancer patients. This information could facilitate the development of preventive strategies to reduce risks and, of course, improve the quality of life for these patients. Additionally, the results of this study may contribute to increased awareness and understanding of the mental and physical health issues of breast cancer patients among healthcare professionals and the general public.

The confidentiality of participating patients was preserved through the anonymization of personal data. Patients were also informed of their right to refuse participation and to withdraw their consent at any time.

### 2.2. Statistical Analysis

The statistical analysis was performed using the JASP software version 0.18.2. Descriptive statistics were computed for both questionnaires. The dependent variables in this study were represented by the scores obtained in each domain. In contrast, the independent variables included age, performance status (ECOG), menopausal status, stage at diagnosis, type of surgery performed (mastectomy/conservative breast surgery), metastasis type, and organ dysfunctions.

We used the Kaplan–Meier survival curve to assess survival probability and the log-rank test to search for statistically significant differences between cancer stage curves. For non-parametric tests, comparing the distribution of questionnaire scores between patient groups, the Mann–Whitney test has been chosen, with significance set at *p* ≤ 0.05.

Multiple linear regression was utilized to explore the associations within a model of seven predictors (age, cancer stage, organ dysfunctions/failures coded as ‘0’ and ‘1’ each) and the scores for every QLQ-C30 and QLQ-BR45 scale. Adjusted R-squared values and standardized coefficient β have been calculated, and significance was determined at *p* ≤ 0.05.

### 2.3. Study Limitations

Similar to any study, ours has inherent limitations. The quality of life of patients was not correlated with the type of treatment administered at the time of questionnaire application; hence, the results stem from patients undergoing diverse therapeutic regimens based on the disease stage and molecular subtype. Additionally, the assessment of life quality before commencing or after completing treatment was not conducted. The questionnaire was administered only once, and as such, we cannot quantify the impact of a specific treatment type on quality of life (QoL).

Quality of life is also influenced by other social variables that were not considered, such as educational level, low income, joblessness, lack of familial support, divorce, the death of a partner, fear of disease progression, or the aggressiveness of treatments.

## 3. Results

**Figure A1:**
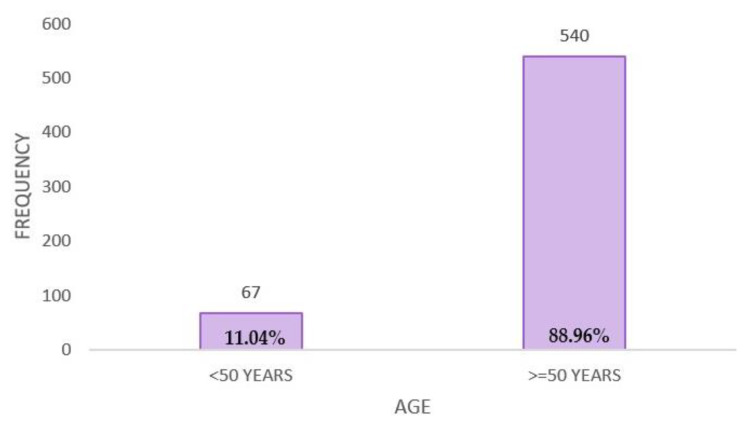
Age distribution.

Following the administration of the two QoL questionnaires, EORTC QLQ-C30 and EORTC QLQ-BR45, we obtained a total of 607 responses for each questionnaire. The mean age of the patients who completed the questionnaires was 64.54 ± 11.60 years, with the group of patients aged <50 years representing 11.04% (*n* = 67) and the group of patients aged ≥50 years representing 88.96% (*n* = 540) (Figure A1).

**Figure A2:**
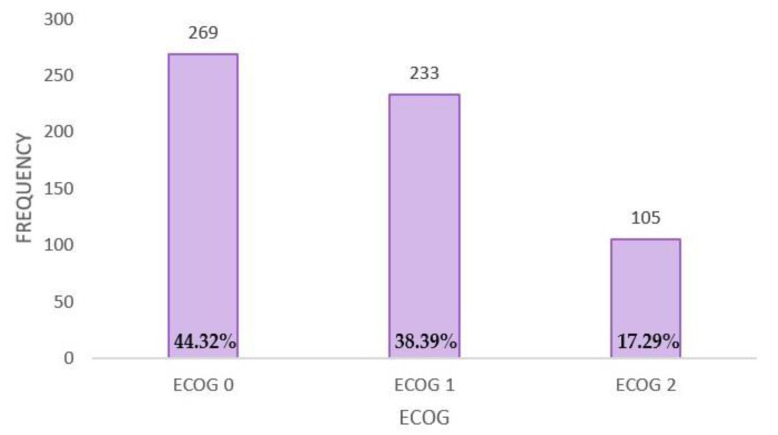
Performance status distribution.

The performance status (ECOG) of eligible patients for this study is distributed as follows: ECOG 0—44.32% of the patient cohort (N = 269); ECOG 1—38.39% of the patient cohort (N = 233); and ECOG 2—17.29% of the patient cohort (N = 105) (Figure A2).

**Figure A3:**
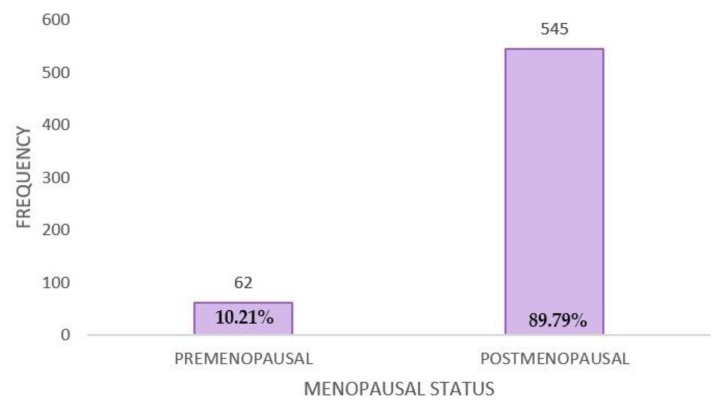
Menopausal status distribution.

Menopausal status was divided into patients in the premenopausal and postmenopausal groups at the time of questionnaire administration. The group of premenopausal patients (N = 62) represented 10.21%, while those in the postmenopausal group (N = 545) represented 89.78% (Figure A3).

**Figure A4:**
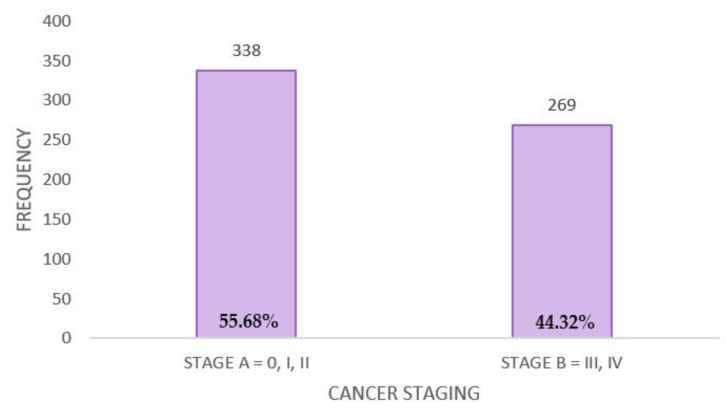
Cancer staging distribution.

Based on the diagnostic stage, two groups were established: stage A, which includes patients diagnosed in stages 0, I, and II (N = 338, representing 55.68%), and stage B, which includes patients diagnosed with stages III and IV (N = 269, representing 44.32%) (Figure A4).

**Figure A5:**
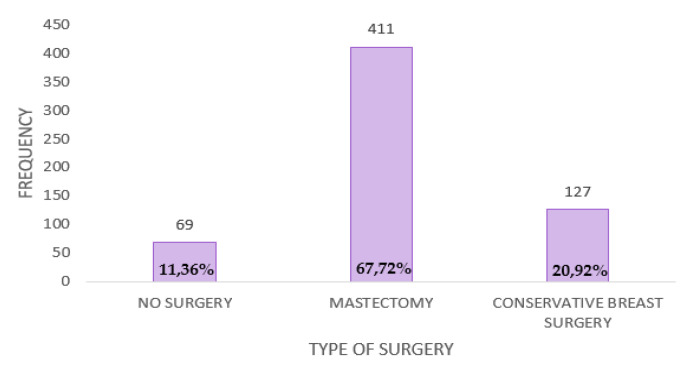
Type of surgery distribution.

Among the patients who responded to the questionnaire, 88.6% (N = 538) underwent surgery. Total mastectomy was performed in 67.71% (N = 411), while 20.92% (N = 127) of them underwent breast-conserving surgery (Figure A5).

**Figure A6:**
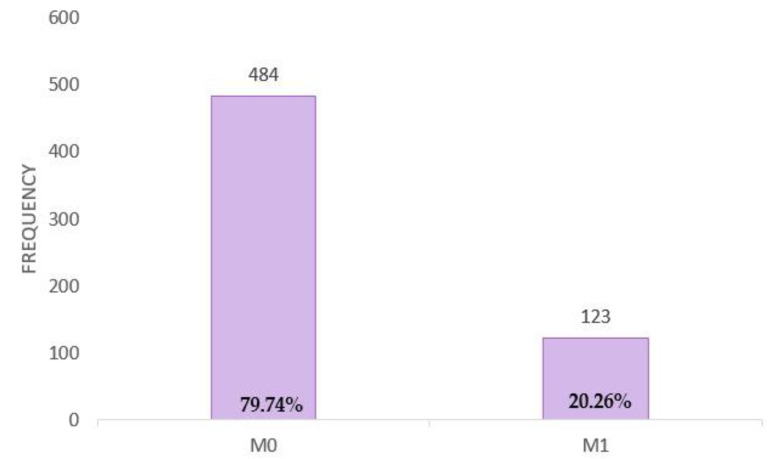
Distribution of patients with and without metastases.

Out of the 607 patients, 79.74% (N = 484) were without metastases, while 20.26% (N = 123) presented with both visceral and non-visceral metastases (Figure A6).

**Figure A7:**
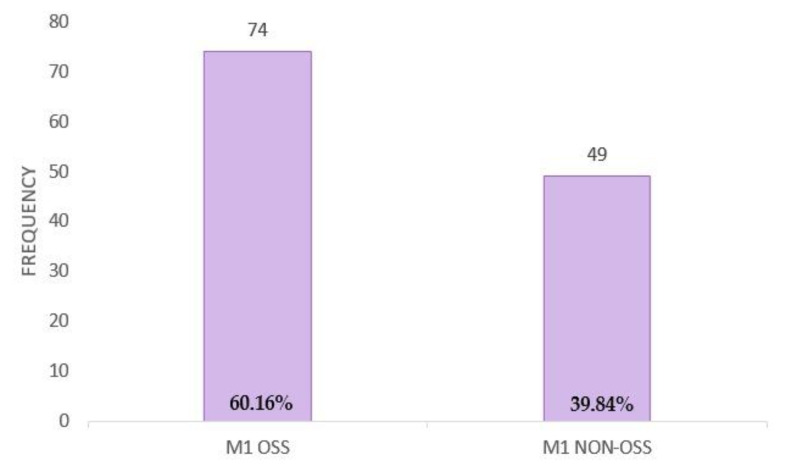
Metastasis distribution.

Among metastatic breast cancer patients, 60.16% (N = 74) were diagnosed only with bone metastases, while 39.84% (N = 49) of them presented non-bone metastases (visceral or non-visceral) (Figure A7).

**Figure A8:**
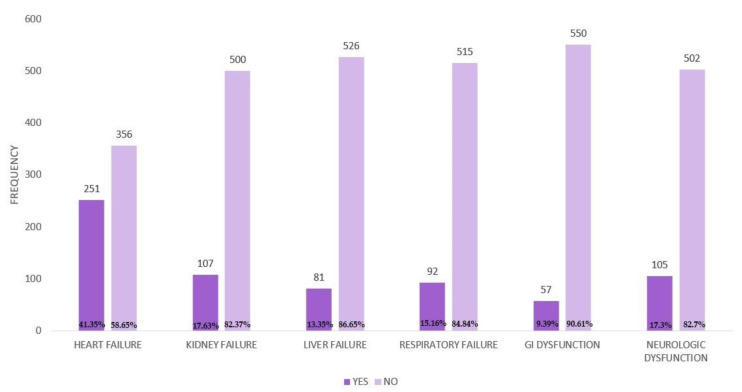
Distribution of organ failure.

We examined patients with organ failure to observe how their quality of life is influenced based on the site of failure. Thus, 41.35% (N = 251) of patients presented heart failure, 17.63% (N = 107) presented kidney failure, 15.16% (N = 92) respiratory failure, 9.39% (N = 57) gastrointestinal dysfunction, and 17.3% (N = 105) neurologic dysfunction (Figure A8).

**Figure 2:**
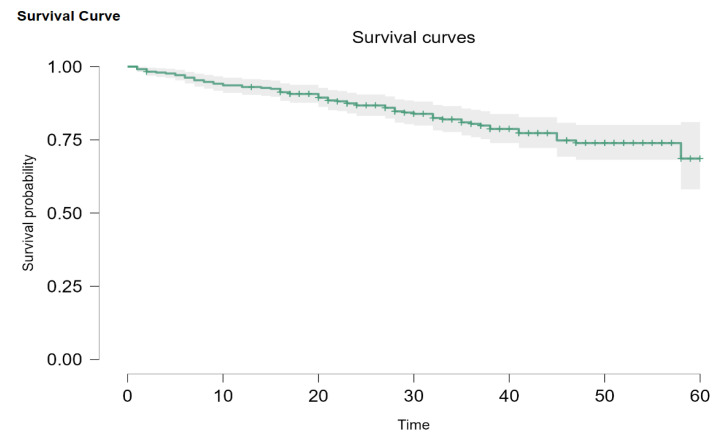
General Kaplan–Meier survival plot.

Regarding the survival rate of our patients, the Kaplan–Meier survival curve was utilized, and the results are presented in Figure 2.

**Figure 3:**
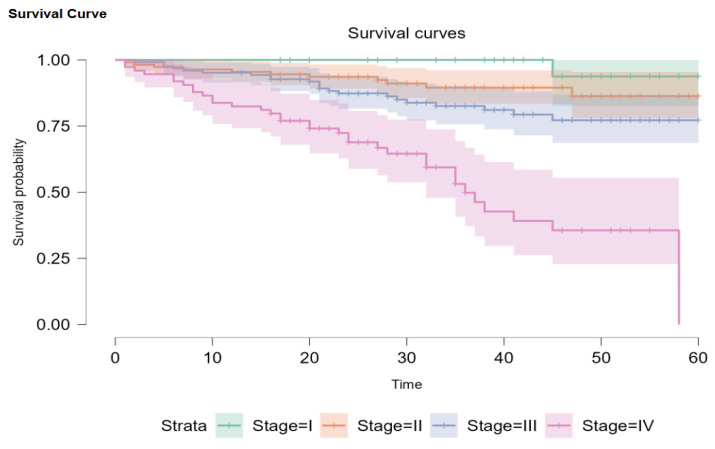
Kaplan–Meier survival plots for disease stages.

Out of our initial patients, only 345 had been newly diagnosed between 1 January 2019 and 1 October 2022. Overall survival probability for every stage was 68.8% (95% CI: 58.1–81.0). Out of 345 patients, 2 (0.5%) had DCIS, 34 (9.8%) were Stage I, 111 (32.2%) were Stage II, 124 (35.9%) were Stage III, and 74 (21.4%) were Stage IV. When divided this way, the survival probability is 93.8% (95% CI: 82.6–100) for Stage I, 86.3% (95% CI: 78.1–95.4) for Stage II disease, and 77.2% (95% CI: 68.7–86.8) for Stage III. Metastatic patients had a 45-month survival probability of 35.6%, with a median survival of 36 months (Figure 3). Due to the Kaplan–Meier product limit estimator, survival at 58 months was 0, and 60-month survival could not be accurately evaluated. According to Carter and Huang [24], survival analysis using the Kaplan–Meier curve is not always reliable for 30 or fewer patients at risk (we had 11 at the 45-month mark) and is too sensitive for single observations in such conditions. The log-rank test showed that there are statistically significant differences between the plots (*p* < 0.001).

The results obtained from assessing the quality of life using the EORTC QLQ-C30 and EORTC QLQ-BR45 questionnaires are presented in Table 1.

In general, on the EORTC QLQ-C30 questionnaire, the global health status displayed a score of 72.18 (on a scale from 0 to 100), indicating that the majority of patients (78.41%) self-evaluated their health and quality of life as good. Additionally, the QLQ-C30 summary score shows similar results. Regarding the functional scales, most patients achieved scores higher than 66.66%, with the highest scores observed in the cognitive functioning (88.30%) and social functioning (89.95%) scales. However, an analysis of the results on the symptoms scale reveals that symptom burden in breast cancer patients arises from insomnia (28.99%), fatigue (13.83%), pain (12.85%), and financial issues (14.99%). Constipation and diarrhea are the least severe symptoms in our patient cohort (9.22% and 2.8% of patients, respectively).

The complementary assessment with the EORTC QLQ BR-45 questionnaire includes three reverse-scoring items: sexual functioning, sexual enjoyment, and breast satisfaction. Higher scores on these scales indicate reduced functionality. The majority of patients are satisfied with their physical appearance (82.53%). In the case of patients who underwent surgery, most were satisfied with the surgical outcome and the appearance of the skin on the affected breast. Despite this, sexual functioning shows a significant impairment—with 93.57% of patients not interested in sexual activity in the last 4 weeks. Only 88 patients were sexually active in the last month, and among them, 67.04% did not experience pleasure during sexual intercourse. Just over half of the surveyed patients (54.69%) express future health-related concerns.

We used the Mann–Whitney test to compare EORTC QLQ-C30 and EORTC QLQ-BR45 score distributions for patients divided by age (under 50 years, over 50 years), menopausal status (premenopausal, postmenopausal), cancer stage (group A, which includes stages 0, I, II, and group B, including stages III or IV), type of surgery (mastectomy v. conservative surgery), organ dysfunction (patients with heart, respiratory, liver, kidney, neurological or gastrointestinal dysfunction v. patients with no dysfunction, respectively), and metastasis location (patients with bone metastasis compared to patients with other loci of metastasis). *p*-values are presented in Table 2, Table 3, Table 4 and Table 5, followed by mean scores with standard deviation (SD) for ease of interpretation.

For QLQ-C30, the age group <50 years, as well as the premenopausal group, obtained better scores in physical functioning (*p* < 0.01 for both) without having other significant results in functional scales. Regarding symptom scales, significant differences were recorded only in appetite loss (*p* = 0.008 and *p* = 0.034, respectively). Overall, the scores of women in the under 50 years and premenopausal group were better than those of women over 50 years and postmenopausal; however, we did not obtain statistically significant results.

Patients in group A (stages 0, I, II) obtained higher results in physical functioning (*p* < 0.01), role functioning (*p* < 0.01), emotional functioning (*p* = 0.013), global health status (*p* = 0.034), and summary score (*p* < 0.01). Additionally, more favorable results were observed in the fatigue (*p* < 0.001), pain (*p* = 0.020), dyspnea (*p* = 0.027), and appetite loss (*p* = 0.038) scores among symptom scales.

Not all patients underwent surgery, so we have a smaller sample size for statistical analysis. However, patients who underwent conservative breast surgery had higher scores in functional scales, with significant differences in physical functioning (*p* = 0.021), role functioning (*p* = 0.005), and summary score (*p* = 0.032). Although overall, patients with conservative breast surgery obtained better scores on symptom scales, the only statistically significant difference was in financial difficulties (*p* = 0.002).

Following the analysis of the quality of life with the QLQ-BR45 questionnaire, on the functional scales, the age group ≥50 years and the postmenopausal group obtained better scores for body image (*p* = 0.022 and *p* = 0.028, respectively) and worse scores for sexual functioning (*p* < 0.001 for both) (sexual functioning is a reversed scoring item). For the future perspective item, patients diagnosed with breast cancer in stages 0, I, and II obtained higher scores (*p* = 0.020). For the group of patients divided by the type of surgery, statistically significant differences were found only on the body image scale (*p* = 0.001).

For symptom scales, patients under 50 years old and those in premenopause recorded higher values on breast symptoms (*p* < 0.001 and *p* = 0.005, respectively) and endocrine sexual symptoms (*p* < 0.001 for both groups) but lower values of scores for skin mucosis symptoms (*p* = 0.007 for both groups). Patients diagnosed with stages III and IV breast cancer obtained higher scores on systemic therapy side effects (*p* = 0.030), arm symptoms (*p* = 0.028), endocrine therapy symptoms (*p* = 0.046), and skin mucosis symptoms (*p* = 0.015). For patients who underwent surgery, statistically significant results were obtained on the arm symptom (*p* = 0.009) and skin mucosis symptom (*p* = 0.006) scales.

Patients with heart failure and breast cancer had a generally worse QoL than patients with no documented heart failure, generally scoring worse on functional and symptom scales from both questionnaires. Statistically significant differences were seen in physical functioning (*p* < 0.001), pain (*p* = 0.034), insomnia (*p* = 0.010), global health status (*p* = 0.024), and summary score (*p* = 0.010) for QLQ-C30 and in sexual functioning (*p* < 0.001), future perspective (*p* = 0.015), breast symptoms (*p* < 0.001), and endocrine sexual symptoms (*p* < 0.001) for QLQ-BR45. For kidney failure patients, there was a similar tendency, scoring generally worse than patients with no documented kidney disease on most items. We identified statistical significance in physical functioning (*p* = 0.018) for QLQ-C30 and body image (*p* = 0.007), sexual functioning (*p* < 0.001), and endocrine sexual symptoms (*p* = 0.009) for QLQ-BR45. Regarding neurologic dysfunction, pain was the sole significant symptom present (*p* = 0.002), as depicted in Table 6.

**Figure 4:**
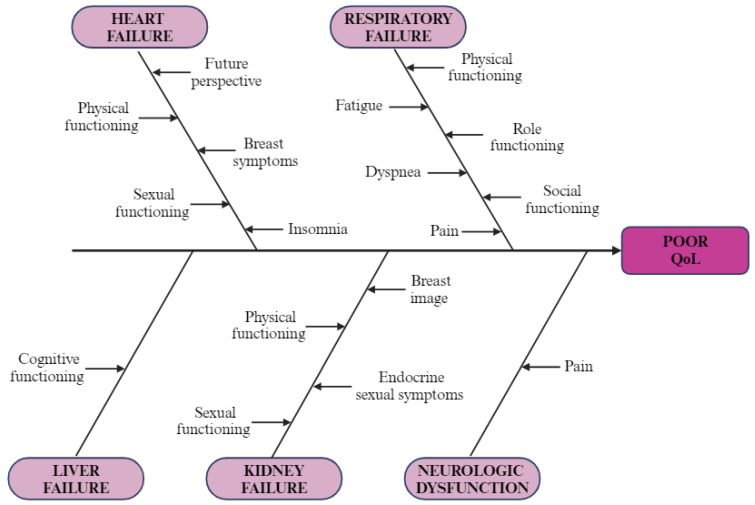
Cause–effect diagram: illustration of negative impact of organ failure on QoL in breast cancer patients (created with BioRender.com (accessed on 9 February 2024)).

Patients with respiratory failure scored worse on every scale from QLQ-C30, with results showing statistical significance for most of them: physical functioning (*p* < 0.001), role functioning (*p* < 0.001), cognitive functioning (*p* = 0.037), social functioning (*p* < 0.001), fatigue (*p* = 0.006), pain (*p* = 0.003), dyspnea (*p* = 0.001), insomnia (*p* = 0.003), appetite loss (*p* = 0.007), constipation (*p* = 0.033), financial difficulties (*p* = 0.035), global health status (*p* = 0.002), and summary score (*p* < 0.001). As for QLQ-BR45, only differences in sexual functioning (*p* = 0.005), future perspective (*p* = 0.011), and endocrine sexual symptoms (*p* = 0.008) were statistically significant, with worse outcomes for the RF+ group. Although liver dysfunction patients scored generally worse on the QLQ-C30 questionnaire, the only statistically significant differences were for cognitive functioning (*p* = 0.018) and summary score (*p* = 0.045) scales. Results for QLQ-BR45 were mixed, as shown in Table 7, with no significant *p*-value. For gastrointestinal dysfunction, no significant changes have been observed. The most relevant results are presented in a cause-effect diagram (Figure 4).

Patients with bone metastases had worse scores for every item on the QLQ-C30 survey except for dyspnea. Statistically significant differences have been reported for physical functioning (*p* = 0.006), pain (*p* = 0.002), global health status (*p* = 0.004), and summary score (*p* = 0.019) scales. Results for QLQ-BR45 are mixed; the only significant difference in M1oss group scoring, as presented in Table 8, is a worse performance in the endocrine therapy symptom (*p* = 0.002) scale. The statistical test yielded an invalid score for the sexual enjoyment scale due to the low number of respondents.

For our multiple linear regression model, we decided to use seven predictors. Age was coded with ‘0’ if <50 years and ‘1’ if ≥50 years. Stage A was labeled as ‘0’ and B as ‘1’. For the rest of the predictors, HF−, KF−, N−, LF−, and RF− groups were marked with ‘0’, while HF+, KF+, N+, LF+, and RF+ were marked with ‘1’. We excluded the GI group from our model in order to make it more efficient, as it seems to not influence scores very much, with no significant *p*-value reported for our previous tests. The menopause predictor was excluded due to overlap with the age predictor. Also, type of surgery and type of metastasis were excluded due to a smaller sample size. One scale from the questionnaires was not valid due to results violating homoscedasticity (NV). Results are shown in Table 9 and Table 10.

Our model’s explanatory power was shown to be statistically significant in multiple scales from both questionnaires (QL2, PF2, RF2, SF, FA, PA, DY, SL, AP, and SS from QLQ-C30 and BI, FU, SX, BR, SM, and ES from QLQ-BR45). The highest degree of variation accounted for by the model was registered for the sexual functioning scale at 21.6% (adjusted R^2^); the predictors that had unfavorably influenced this score were primarily older age (standardized coefficient ꞵ = 0.364, *p* < 0.001) and heart failure (ꞵ = 0.192, *p* < 0.001).

The model accounts for around 1–3% of variation in most statistically significant results. We report higher adjusted R^2^ for PF2 (0.097), RF2 (0.041), SS (0.044), and ES (0.065). For the summary score of QLQ-C30 (SS), the most important predictor was respiratory failure (ꞵ = −0.142, *p* < 0.001), while heart failure had the highest influence on PF2 (ꞵ = −0.206, *p* < 0.001) and tumor stage influenced RF2 (ꞵ = −0.138, *p* < 0.001). Endocrine sexual symptoms were lower in the ≥ 50 age group, thus being the most influential predictor for the scale (ꞵ= −0.189, *p* < 0.001).

## 4. Discussion

### 4.1. Survival Analysis

Our study shows a poorer 5-year survival rate than data from the Concorde-3 study for Romania (74.8 5-year net survival rate) [25]. Differences between 5-year survival rates could be explained by either a smaller population lot (345 vs. 2205 patients) or different geographic regions (Bucharest vs. Cluj). The SARS-CoV-2 pandemic might have been an influencing factor as Concorde-3 data are from 2010 to 2014, compared to data registered between 2019 and 2022 in our case. We also register a lower overall survival rate than Western European countries (86% in Italy, 85.2% in Spain, and 88.8% in Sweden) [25].

If divided by stages, our results seem to be similar to other studies from Europe (Italy, Spain) [26,27]. This might suggest that patients in our center seem to receive adequate treatment, but they are treated at a more advanced stage, as 21.4% of the patients included in our survival analysis were metastatic compared to only 3.8% in Mangone et al. and 6.8% in Pascual et al. [26,27].

### 4.2. Analysis of QoL Results Using EORTC QLQ-C30 and EORTC QLQ-BR45 Questionnaires

Our study presents the scores of global health status (72.18%) and summary scores as high scores, which suggests a good quality of life. Also, in functional scales, we obtained high scores as well, but results from the symptom scale indicate a negative impact on QoL. Imran et al. collected similar results in both the overall health scores and the symptom scale. The most distressing symptom in our study was insomnia, affecting 28.99% of patients; followed by fatigue, which affected 13.83%; and then pain, affecting 12.85% of patients [28]. In their study, Jassim et al. obtained a result of 63.9 (95% CI 61.21–66.66) for overall health scores, suggesting a favorable quality of life, but lower compared to our study [29]. The highest score was 77.5 for functional scales, and the lowest was 63.4 for emotional function. However, the most distressing symptom in their study was fatigue, followed by hair loss. The presence of metastases and mastectomy were associated with impairment across all questionnaire scales, contributing to a significant reduction in the quality of life of patients diagnosed with breast cancer. Sexual dysfunction posed a real problem in their patient cohort. Similarly, in our study, a significant issue regarding sexual functioning was observed. The score values indicate that patients were not interested in sexual activity in the last month, and sexually active patients faced sexual-related issues [30]. Castillo et al. highlighted in a review that physicians often do not discuss sexual functioning with breast cancer patients; hence, sexual dysfunction remains an underdiagnosed and undertreated entity in breast cancer patients [31]. Causes leading to sexual dysfunction include psychological factors related to breast cancer, on the one hand, and, on the other hand, the side effects of therapy [31].

Therefore, we compared the quality of life in breast cancer patients by dividing the group based on several parameters such as age at the time of survey administration, menopausal status, diagnostic stage, and the type of surgery performed.

### 4.3. Age and Menopausal Status

The evaluation of the results reveals that we identified several similarities between patient groups divided by age and menopausal status. This finding can be connected with the chosen age limit for dividing the group being close to the onset of menopause. Regarding functional scales, our study showed that age < 50 years and premenopausal status have a positive impact on physical functioning (*p* < 0.01 for both). At the same time, these same patient groups are more affected from the perspective of body image (*p* = 0.022 and *p* = 0.028, respectively) compared to patients over 50 years old or those in menopause. In the case of patients under 50 years old and in premenopause, our study showed that QoL is negatively influenced, especially by breast symptoms (*p* < 0.001 and *p* = 0.005, respectively) and endocrine sexual symptoms (*p* < 0.001 for both groups). According to our linear regression model, the most significant predictor of endocrine sexual symptoms has been age; symptoms have been more severe in patients under age 50. Manifestations in the appetite loss category (*p* = 0.008 and *p* = 0.034) and skin mucosis symptoms (*p* = 0.007 for both groups) are more important for the quality of life of patients over 50 years old or in menopause. Marschner et al. conducted an extensive examination of QoL for patients in pre- and post-menopause receiving systemic treatment [32]. The study included breast cancer patients, with 251 patients in premenopause and 478 in postmenopause, who completed various QoL questionnaires: BFI, FACT-G, FACT-ES, FACT-Taxane, HADS, and EORTC QLQ-BR23. They concluded that in recent years, there has been a significant improvement in the overall quality of life for this category of patients, but different parameters need to be monitored based on menopausal status. Thus, premenopausal patients require special attention for emotional issues, anxiety, body image, and endocrine symptoms [33]. In contrast, postmenopausal patients need special care as they reported a decrease in social and familial support. Moreover, the latter reported late adverse reactions, neurotoxicity, and endocrine symptoms. Therefore, we believe it is essential for the oncologist to discuss with the patient and identify those areas that require special attention based on age or menopausal status, both during treatment and throughout the follow-up process.

### 4.4. Cancer Staging

Our study indicates that the QoL is influenced by the diagnostic stage of patients with breast cancer. The results show that QoL was negatively affected in the group of patients diagnosed with breast cancer stage III or IV, with statistically significant differences recorded in physical functioning (*p* < 0.01), role functioning (*p* < 0.01), emotional functioning (*p* = 0.013), global health score (*p* = 0.034), and summary score (*p* < 0.01). Our results regarding the linear regression emphasized that the most important predictor of role functioning has been the stage at diagnosis (ꞵ= −0.138, *p* < 0.001).

Furthermore, for patients with advanced forms of the disease, fatigue (*p* < 0.001), pain (*p* = 0.020), dyspnea (*p* = 0.027), and lack of appetite (*p* = 0.038) were the main factors included in QLQ-C30 that contributed to symptom burden. It is important to note that patients in Group B recorded a higher degree of health-related concerns; hence, patients are aware of the advanced stage of the disease, and they are afraid of the progression of the disease and the severity of adverse reactions to systemic treatments.

Consistent with these concerns, scores for symptom scales in the QLQ-BR45 questionnaire indicate a greater impact of systemic therapy side effects (*p* = 0.030), endocrine therapy symptoms (*p* = 0.046), arm symptoms (*p* = 0.028), and skin mucosis symptoms (*p* = 0.015) over the quality of life of breast cancer patients in stage III and IV. De Mello Ramirez Medina et al. evaluated the link between clinical stage and health-related quality of life (HRQOL) in their study [34]. They discovered that early breast cancer stage was associated with better role functioning; in contrast, advanced breast cancer registered worse results in role functioning score (*p* = 0.029). According to the abovementioned study, King et al. promote the idea that an early diagnosis of cancer is not associated with changes in physical functioning, role functioning, and emotional functioning. However, chemotherapy can deeply affect the QoL [35]. Broeckel et al., Jacobsen et al., and Weitzner et al. have reported chemotherapy as a factor that could predict a worsening of the quality of life [36,37,38]. Yen et al. found similar results, in which radiotherapy was strongly associated with a deterioration of the quality of life [39]. These results strengthen the importance of early diagnosis of breast cancer to maintain an optimal quality of life.

### 4.5. Type of Surgery

Regarding patients who have undergone surgery, our study highlights that patients for whom mastectomy was performed experience poor physical and role functioning. As a result, they reflect a reduced summary score, which indicates a worsening in QoL compared to patients who have undergone conservative breast surgery (*p* = 0.032). Therefore, studying the QoL in mastectomized patients can improve their lives. The oncologist should have the responsibility of identifying those variables that can negatively impact the QoL of these patients. For example, Akça et al. emphasized in their work that mastectomy led to a reduction in global QoL (*p* < 0.001) and social functionality (*p* < 0.01), while fatigue and constipation scores have been improved in conservative surgery [40]. De Ligt et al. reinforce the observation with their results: 92% of patients with a mastectomy had three or more symptoms, such as fatigue, pain, dyspnea, and insomnia, in the first years after mastectomy [41]. However, insomnia was the main symptom in 62% of survivors through their 5-year survivorship. Moreover, Schmidt et al. argue in their study that fatigue could be observed even 10 years post-mastectomy [42]. In the context of the symptom scale QLQ-C30, patients with mastectomy presented financial difficulties (*p* = 0.002) in contrast with the conservative breast approach. The rationale could be due to the additional cost of therapy, medication, and recovery procedures, as well as absenteeism from work due to the disease. Moreover, emotional instability may lead to depressive symptoms and therefore lead to financial difficulties. In 2017, Kim et al. showed that the incidence of depression persists 3 years after a mastectomy has been performed [43]. The evidence underscores the need for comprehensive care that addresses not just the physical but also the psychological aspects of recovery.

Regarding QLQ-BR45, our study showed that body image, sexual functioning, arm symptoms, and skin mucosis symptoms had been significantly impaired in mastectomized patients. Using QLQ-BR23, Akça et al. gathered similar results to our study. These results emphasize that body image is less altered in patients who have undergone conservative breast surgery. Additionally, they correlated the perception of body image and hair loss, lymphedema, and breast symptoms. Therefore, a complex linkage exists between physical health, mental well-being, and body image in patients undergoing surgical treatment [40]. Sexual function and sexual enjoyment have also been evaluated as being significantly impaired after mastectomy. Two studies conducted by Aerts et al. and Molavi et al. compared sexual functioning and sexual satisfaction in patients who underwent mastectomy versus conservative surgery [44,45]. Similar to our study, their results showed that mastectomy is a significant factor for sexual dysfunction in patients with breast cancer. Thus, treatment decisions and post-operative care and support highlight the potential impact on sexual health.

In contrast to mastectomy, mammary reconstruction could represent a method to alleviate sexual functioning, as presented in the work of Neto et al. [46]. Arm symptoms such as arm pain, shoulder pain, lymphedema, and limited arm mobility are frequently present in our patients after mastectomy. Nesvold et al. point out in their work the late effect on the arm and shoulder in patients with stage II breast cancer who underwent mastectomy. They observed that arm/shoulder symptoms are more frequently encountered in patients treated with radical mastectomy. The authors also mention the importance of early identification of these symptoms to prevent late complications at the arm/shoulder level. Thus, the long-term outcome after radical mastectomy should be carefully monitored [47].

### 4.6. Quality of Life of Patients with Breast Cancer and Organ Failure

Another significant aspect of breast cancer patients is the presence of multiple comorbidities. One of the most severe is organ dysfunction, which can severely impact the QoL. Some of these functional impairments were already pre-existing before the diagnosis of the neoplastic disease, while other organ functions have been altered due to disease progression or therapeutic side effects such as heart failure after trastuzumab [48].

#### 4.6.1. Heart Failure

Patients with heart failure had a worsening in QoL than patients with no documented heart failure; functional and symptom scales from both EORTC QLQ-C30 and EORTC QLQ-BR45 have been broadly reduced. Our linear regression model showed that heart failure significantly impaired physical functioning (ꞵ = −0.206, *p* < 0.001). Therefore, QoL in breast cancer patients and heart failure is reduced primarily due to a worsening in the physical function sphere (*p* < 0.001). Additionally, poor performance in global health status (*p* = 0.024) and QLQ-C30 summary score (*p* = 0.010) have been noticed in our patients. Regarding QLQ-BR45, our study presents the negative impact of sexual functioning (*p* < 0.001), future perspective (*p* = 0.015), breast symptoms (<0.001), and endocrine sexual symptoms (*p* < 0.001) on QoL in heart failure patients. Ghuloom et al. conducted a meta-analysis encompassing 29 studies from the literature, which examined the quality of life of patients with heart failure [49]. They concluded that women who have heart failure experience a poorer overall quality of life compared to men. In addition, Schultz et al. presented the fact that widowed patients experienced more cardiovascular events and heart failure complications compared to married patients [50]. These results are supported by Tromp et al., who obtained similar results in their study, demonstrating that female patients with heart failure and breast cancer who did not have a partner at the time of the study (unmarried, divorced, widowed) had a reduced quality of life compared to those who were married (*p* < 0.001). These studies underscore the importance of family and social support in maintaining a good quality of life [51].

#### 4.6.2. Renal Failure

With regard to breast cancer patients and renal failure, patients with worse scores in physical functioning (*p* = 0.018) had a decline in QoL compared to patients without renal failure. As expected, the systemic impact of renal failure on the body’s homeostasis has a deep effect on the person’s performance. Edema, reduced heart function, and dialysis are several consequences that reduce the ability to perform activities such as long and short walks and doing groceries. In addition, Cai et al. emphasize that dialysis also represents a factor that disturbs the dose adjustments, as well as the schedule between the administration of the chemotherapeutic drug and dialysis [52]. Renal failure is a severe comorbidity in breast cancer patients, requiring dose adjustments for chemotherapy regimens based on creatinine clearance [53]. Currently, there are very few studies in the literature on the treatment of breast cancer in patients with end-stage renal disease. Modi et al. concluded in their study that these patients rarely successfully complete standard neoadjuvant chemotherapy, adjuvant chemotherapy, or endocrine therapy [54].

Regarding QLQ-BR45, the quality of life for breast cancer patients with renal failure has been affected by aspects such as body image (*p* = 0.007), sexual functioning (*p* < 0.001), and endocrine sexual symptoms (*p* = 0.009). Deshpande et al. emphasized in their study that chronic diseases, including chronic kidney disease, must be closely monitored not only at the diagnosis and during breast cancer treatment but also integrated as continuous surveillance [55]. This necessitates collaboration between oncologists, surgeons, and primary care physicians to improve physical and social functioning.

#### 4.6.3. Neurologic Dysfunction

In the context of neurologic dysfunction, our study explored that QoL has been influenced only by the pain scale (*p* = 0.002). Neurologic patients represent a fragile population, especially in breast cancer patients. As presented by Ju et al., spine metastases account for ⅔ of osseous dissemination [56]. Patients may suffer from pain refractory to treatment and neurological dysfunction, symptoms which can reduce their quality of life. Therefore, the main goal of treatment is to improve spinal stability, relieve pain, and reduce neurological dysfunction. Quraishi et al. emphasize the importance of a multidisciplinary approach in metastatic spinal tumors, in which the oncologist, hematologist, histopathologist, spinal surgeon, and radiologist cooperate to provide the best care for the patient [57]. Moreover, Zoccali et al. mention in their work that the advances in targeted therapy of breast cancer have improved the 10-year survival, and therefore, the actual survival rate prediction accuracy in breast cancer with neurological dysfunction is diminishing, calling for a need for new techniques [58].

On the other hand, the treatments undergone by breast cancer patients can have an impact on neurological function due to neurotoxicity phenomena. These influence the quality of life of oncology patients, especially concerning cognitive impairment, fatigue, or long-term pain, as reported by Lacourt and Heijnen in their study [59]. For breast cancer patients treated with adjuvant chemotherapy, neuroimaging studies have been conducted, revealing a range of structural and functional alterations in the brain, including changes in grey matter volume and density [60,61,62]. This could explain the cognitive deficits faced by breast cancer patients. Additionally, the impact of chemotherapy-induced peripheral neuropathy on QoL should not be overlooked. Faruqi et al. report in their study that taxane-based chemotherapy-induced peripheral neuropathy (docetaxel, paclitaxel) can persist up to 3 years after the completion of treatment [63]. Nilsson et al. observed that patients with a history of breast cancer have a higher risk of experiencing a stroke without being able to specify whether this risk is associated with the neoplasm or the treatments undergone by the patient [64].

Impairment of neurological function is often associated with manifestations in the psychiatric domain, especially in the case of breast cancer patients. Anxiety and depression are certainly the most common problems faced by these patients; depression is frequently underdiagnosed and undertreated despite its significant impact on quality of life, treatment adherence, and survival [65]. External support from a psychologist, psychiatrist, family, and other social supports should be implemented and maintained during the whole oncologic surveillance and after. Additionally, internal coping mechanisms could represent another explanation for the absence of other changes in functional and symptom scores. The way the patient thinks about the disease and what the current possibilities are that could aid in managing the diagnosis and the treatment could significantly improve the QoL in this particular population. In their research, Guerreiro Goddoy et al. expose that pain in breast cancer patients has been managed using physical, psychological, religious, and environmental coping mechanisms. Painkillers, resting, religious services, talking to friends and family, and heat and cold exposure were several ways of limiting the pain and thus increasing the quality of life [66]. Additionally, Brown T et al. accentuate the need for nurses dedicated to breast cancer patients. Their supportive role could correctly identify the large sphere of needs, from physical, psychological, social, and sexual to cultural and spiritual beliefs. Therefore, pain remains a significant factor that should be targeted in this category, as patients could present a reduced tolerance to pain as a result of neurological dysfunction [67].

#### 4.6.4. Liver Failure

Lower cognitive functioning scores (*p* = 0.018) have been noticed in patients with liver failure. As hepatic function tends to deteriorate gradually in this category of patients and metabolites start to accumulate, cognitive difficulties may occur [68]. Pinto et al. expose in their work the challenges that come in the context of cognitive functioning, as they found no correlation between symptoms such as fatigue, anxiety, depression, and neurological impairment in cognitive tests [69]. Etiology is still undiscovered, and several mechanisms significant to liver failure have been proposed: oxidative damage, hormonal changes, immune dysregulations, and hypercoagulation [70]. In addition, while we are able to assess cognitive function, no significant intervention to help alleviate it has been proposed [71]. Moreover, a reduced QLQ-C30 summary score observed in our study (*p* = 0.045) could provide insight into the early detection of hepatic dysfunction due to the progressive nature of the adverse reaction after chemotherapeutic regimens [72]. Ang et al. present in their work that tamoxifen could lead to the development of non-alcoholic steatohepatitis, while gemcitabine has been associated with liver fibrosis [73]. Quinten et al. emphasize the importance of health-related quality of life (HRQOL). They presented that EORTC QLQ-C30 strongly supports its value as a prognostic indicator, in which sociodemographic and clinical factors better predicted the survival rate of cancer patients. Therefore, changes in HRQOL during oncologic treatment could bring more information about the patient’s prognostic [74]. However, no changes in QLQ-BR45 scores have been observed in our patients.

#### 4.6.5. Respiratory Failure

In breast cancer patients, both respiratory and cardiac failure significantly impact the quality of life. The quality of life of patients with breast cancer and respiratory failure has been particularly affected on functional scales. Our patients have exhibited a decline in physical functioning (*p* < 0.001), role functioning (*p* < 0.001), cognitive functioning (*p* = 0.037), and social functioning (*p* < 0.001) scores. According to the linear regression model, the most critical variable for the outcome of the QLQ-C30 summary score was respiratory failure (ꞵ = −0.142, *p* < 0.001).

As expected, the reduction in oxygen delivery–supply balance severely impacts breast cancer patients. This is translated into a poor capacity to perform daily activities and engage in the community. Due to its local and systemic effect, cancer therapy represents one of the major causes of respiratory failure. Surgical treatment [75], such as radical mastectomy [76]—which reduces thoracic mobility and affects body posture—and chest radiation—which may diminish exercise capacity by reducing maximal oxygen consumption—can trigger the development of respiratory failure. Additionally, Suesada et al. suggested that respiratory capacity may worsen due to thoracic radiotherapy and the restriction of chest wall mobility [77]. Ciesla et al. [78] have shown that the parenchymal of the lung may be damaged due to radiation, while Santos et al. encompass these results by describing the injury of type II pneumocytes, surfactant, and basement membrane [79]. Additionally, Curigliano et al. have observed that hormonal therapy, such as tamoxifen, may increase pulmonary toxicity and therefore decrease pulmonary function [80]. A worsening in global health status (*p* = 0.002) and QLQ-30 summary (*p* < 0.001) scores encompass all the disease burdens that this category of patients has to face. Additionally, the respiratory system is acknowledged to be one of the first organs that can succumb, promoting the onset of multiple organ failure [81]. Therefore, these patients necessitate careful monitoring during their treatment and post-therapy due to its considerable impairment of the patient because long-term QoL has been observed to be altered by acute respiratory distress syndrome (ARDS) and mechanical ventilation [33]. Additionally, other symptoms have been significantly present in patients with respiratory failure: fatigue (*p* = 0.006), pain (*p* = 0.003), dyspnea (*p* = 0.001), insomnia (*p* = 0.003), appetite loss (*p* = 0.007), constipation (*p* = 0.033), and financial difficulties (0.035). Regarding QLQ-BR45, sexual functioning (*p* = 0.005), future perspective (*p* = 0.011), and endocrine sexual symptoms (*p* = 0.008) had a worsening in scores. Notable is the future perspective on the personal outcome. Respiratory failure often leads to increased anxiety, resulting in a sense of despair. As such, it is crucial to establish and sustain support from psychologists, psychiatrists, family, and other social networks throughout the entire period of cancer monitoring. Kim et al. conclude in their work that a nurse-led psychological intervention can profoundly impact symptoms such as anxiety and depression and improve global health status and physical and emotional functioning [82]. Therefore, the need for such nurses represents an important pillar in improving future perspectives and reducing psychological distress.

### 4.7. Patients with Osseous and Non-Osseous Metastases

In terms of osseous and non-osseous metastases, our results are similar to the general expectations found in the literature. Patients with osseous metastases had a lower QoL mainly due to two factors: physical functioning (*p* = 0.006) and pain symptoms (*p* = 0.002). These results are in agreement with the reduced scores obtained through the QLQ-C30 questionnaire: global health status score (*p* = 0.004) and summary score (*p* = 0.019). Our findings highlight the impact that bone metastases, which are inherently extremely painful, can have on patients with metastatic breast cancer. In terms of QLQ-BR45, the only significant change was observed in symptoms related to endocrine therapy (*p* = 0.002). Moreover, our study demonstrates that hormonal therapy-related symptoms (musculoskeletal symptoms, mood swings, dizziness, weight gain, hot flashes) represent a significant factor influencing the quality of life in patients with metastatic breast cancer. It is crucial for oncologists to identify these adverse effects associated with endocrine therapy. Franzoi et al. provide a review of pharmacological and non-pharmacological methods through which adverse reactions can be effectively managed [83]. The main aim of these methods is to improve the quality of life and treatment adherence for patients undergoing endocrine therapy. Neelam Sharma et al. conducted a study on factors affecting the quality of life among breast cancer patients [84]. They observed that the lowest scores were recorded in patients with metastatic disease. Their study results were similar to ours, showing impairment in global health status, fatigue, pain, and role functioning. Chao et al. found comparable results in patients with osseous metastatic disease at the end of therapy [85]. Their study results indicate that QoL was affected in terms of physical functioning, role functioning, emotional functioning, and pain in the case of patients with breast cancer and bone metastases. Guinan et al. encompass these results with their patients scoring lower in physical functioning, role functioning, emotional functioning, and pain [86].

## 5. Conclusions

This study set out to evaluate the QoL in breast cancer patients in the context of organ failures and osseous metastases. The second aim of this investigation was to assess the survival curve to understand their clinical pathway and quality of life. To the best of our knowledge, this is the first comprehensive investigation with a sufficiently large patient cohort and, thus, fills a gap in the specialized literature related to QoL in patients diagnosed with breast cancer in Romania.

Our work has found that age under 50 years and premenopausal status correspond to factors with a beneficial influence over physical functioning; on the other hand, for this group of patients, body image had a significant impact on QoL in comparison with patients over 50 years or in menopause. QoL for patients under 50 years and those in premenopause was altered by breast symptoms and endocrine sexual symptoms. Additionally, as we expected, breast cancer patients in stages III and IV presented a lower quality of life in contrast with the women diagnosed in the early stages. The primary aspects that enhanced the symptom burden for patients with advanced-stage disease were fatigue, pain, dyspnea, and lack of appetite. Regarding the type of surgery performed, patients who underwent mastectomy experienced difficulties in physical functioning and role functioning compared to those who had conservative breast surgery. Moreover, mastectomy was associated with a significant decrease in quality of life, affecting body image and sexual functioning, while patients who had a conservative surgical intervention achieved better results for arm symptoms and skin mucosis symptoms.

In the context of organ failures, this approach will prove useful in expanding our understanding of how the dysfunction of an organ influences the quality of life in breast cancer patients. Our results indicate that patients with breast cancer and heart failure experienced a lower QoL, with significant impairment in physical functioning, sexual functioning, and future perspective compared to patients with malignancy and without cardiac insufficiency. Renal failure has been identified to negatively impact physical functioning, body image, and endocrine sexual symptoms. Patients with liver failure have been identified with lower scores on the cognitive functional scales. Furthermore, respiratory failure led to a significant decrease in physical, role, cognitive, and social functioning, while the most frequent symptoms have been dyspnea, fatigue, pain, and financial issues.

Analyzing the quality of life based on metastatic sites, we have registered a lower quality of life in the case of patients with bone metastasis, especially because of debilitation on physical functioning and pain scales. These aspects are often observed in clinical practice; thus, we expected this outcome.

Regarding survival rate, the patients in our study had a 5-year overall life expectancy of 68.8%. Upon stratification by stages, the likelihood of survival was observed to be 93.8% for Stage I, 86.3% for Stage II, and 77.2% for Stage III. For patients with metastatic disease, the probability of survival over a period of 45 months was found to be 35.6%, with a median survival duration of 36 months.

Considerably more work will need to be carried out to introduce the evaluation of the QoL in clinical practice through standardized instruments from the first presentation of the breast cancer patient to the oncologist and throughout the entire follow-up process. Further studies and clinical trials need to be carried out in order to assess the QoL in the context of organ failures, which will provide insight regarding the administration of pharmacological and non-pharmacological interventions to alleviate the quality of life, raising adherence to treatment and global survival of patients diagnosed with breast cancer.

## Figures and Tables

**Table 1:** The assessment of QoL in breast cancer patients by using EORTC QLQ-C30 and EORTC QLQ-BR45.

Items	N	Median	Mean	95% Confidence Interval Mean		Std. Deviation	N (%) Score < 33.33%	N (%) Score > 66.66%
QLQ-C30								
Functional scales								
Physical functioning	607	80.000	73.465	71.824–75.106		20.589	38 (6.26%)	445 (73.31%)
Role functioning	607	83.333	74.602	72.394–76.810		27.696	113 (18.61%)	473 (77.92%)
Emotional functional	607	83.333	77.732	75.969–79.495		22.115	52 (8.56%)	488 (80.39%)
Cognitive functioning	607	83.333	82.345	80.676–84.013		20.932	37 (6.09%)	536 (88.30%)
Social functioning	607	100.000	89.154	87.419–90.890		21.770	49 (8.07%)	546 (89.95%)
Symptoms scales								
Fatigue	607	22.222	28.263	26.322–30.204		24.351	440 (72.48%)	84 (13.83%)
Nausea and vomiting	607	0.000	10.983	9.164–12.802		22.823	584 (96.21%)	14 (2.3%)
Pain	607	16.667	23.833	21.751–25.915		26.123	477 (78.58%)	78 (12.85%)
Dyspnea	607	0.000	11.148	9.351–12.944		22.541	556 (91.59%)	51 (8.4%)
Insomnia	607	33.333	32.070	29.700–34.440		29.732	431 (71%)	176 (28.99%)
Appetite loss	607	0.000	10.983	9.164–12.802		22.823	576 (94.89%)	61 (10.04%)
Constipation	607	0.000	12.850	11.058–14.643		22.487	551 (90.77%)	56 (9.22%)
Diarrhea	607	0.000	4.448	3.325–5.571		14.084	590 (97.19%)	17(2.8%)
Financial difficulties	607	0.000	19.110	16.999–21.221		26.482	516 (85%)	91 (14.99%)
Global health status/QoL	607	75.000	72.186	70.698–73.673		18.664	33 (5.4%)	476 (78.41%)
QLQ-C30 summary score	607	85.385	82.179	81.030–83.327		14.409	4 (0.65%)	521 (85.83%)
EORTC QLQ BR-45								
Functional scales								
Body image	607	100.000	83.114	81.135–85.092		24.824	56 (9.22%)	501 (82.53%)
Sexual functioning *	607	100.000	92.724	91.291–94.157		17.981	30 (4.9%)	568 (93.57%)
Sexual enjoyment *	88	33.333	43.939	38.634–49.245		17.981	59 (67.04%)	29 (32.95%)
Future perspective	607	66.667	56.013	53.287–58.739		34.202	275 (45.30%)	332 (54.69%)
Breast satisfaction *	539	0.000	16.759	14.800–18.719		23.161	486 (90.16%)	37 (6.86%)
Symptoms scales								
Systemic therapy side effect	607	14.286	18.655	17.388–19.923		15.900	525 (86.49%)	4 (0.65%)
Breast Symptoms	607	8.3333	11.093	10.039–12.146		13.217	583 (96.04%)	2 (0.32%)
Arm symptoms	607	11.111	18.964	17.327–20.601		20.535	504 (83.03%)	36 (5.93%)
Upset by hair loss	199	33.333	28.978	24.503–33.453		32.011	145 (72.86%)	54 (27.13%)
Endocrine therapy symptoms	607	20.000	21.087	19.853–22.321		15.479	502 (82.70%)	5 (0.82%)
Skin mucosis symptoms	607	11.111	14.488	13.402–15.574		13.625	568 (93.57%)	1 (0.16%)
Endocrine sexual symptoms	607	0.000	9.253	7.791–10.715		18.341	561 (92.42%)	21 (3.45%)

A higher score for functional scales means better functioning. Scoring < 33.33% suggests a significative impairment of QoL, while scoring > 66.66% indicates an improved QoL. A higher score on symptom scales means worse functioning. Scoring < 33.33% suggests a lower symptom burden, and scoring > 66.66% indicates a significative symptom burden. Sexual enjoyment—applicable only for sexually active patients in the last month. Upset by hair loss—applicable only for patients who have observed hair loss in the last week. Breast satisfaction—applicable only for patients who underwent the surgery. * reverse scoring items

**Table 2:** The analysis of variables in functional scales, global health status, and QLQ-C30 summary score in EORTC QLQ-C30.

Variables	PF2 Mean (SD)	RF2 Mean (SD)	EF Mean (SD)	CF Mean (SD)	SF Mean (SD)	QL2 Mean (SD)	QLQ-C30 SS Mean (SD)
Age							
<50 years (N = 67)	81.99 (15.99)	76.36 (28.74)	74.75 (20.61)	84.08 (21.79)	88.06 (23.17)	73.75 (18.13)	85.31 (10.59)
≥50 years (N = 540)	72.40 (20.85)	74.38 (27.58)	78.10 (22.28)	82.130 (20.83)	89.29 (21.60)	71.99 (18.73)	81.79 (14.77)
*p*-value	**<0.01**	0.437	0.084	0.273	0.681	0.523	0.170
Menopausal status							
Premenopausal (N = 62)	81.613 (16.538)	76.61 (29.01)	74.32 (21.30)	83.60 (21.65)	87.09 (24.59)	72.44 (18.94)	84.99 (11.25)
Menopausal (N = 545)	72.53 (20.81)	74.37 (27.56)	78.11 (22.19)	82.20 (20.89)	89.38 (21.43)	72.15 (18.64)	81.85 (14.70)
*p*-value	**<0.01**	0.390	0.084	0.463	0.681	0.938	0.220
Cancer staging							
Stage A = 0, I, II (N = 338)	76.11 (19.28)	78.69 (25.83)	79.14 (22.39)	83.08 (21.03)	90.43 (20.63)	73.81 (17.64)	83.88 (14.11)
Stage B = III, IV (N = 269)	70.13 (21.69)	69.45 (29.10)	75.96 (21.66)	81.41 (20.80)	87.54 (23.05)	70.13 (19.71)	80.04 (14.51)
*p*-value	**<0.01**	**<0.01**	**0.013**	0.180	0.074	**0.034**	**<0.01**
Type of surgery							
Mastectomy (N = 411)	73.26 (20.45)	74.16 (28.22)	77.63 (22.32)	82.48 (21.33)	89.05 (22.52)	72.66 (17.80)	82.21 (14.29)
Conservative breast surgery (N = 127)	78.11 (18.87)	82.80 (21.91)	81.43 (20.73)	83.33 (19.13)	91.33 (17.79)	76.24 (18.00)	85.24 (12.75)
*p*-value	**0.021**	**0.005**	0.051	0.923	0.676	0.079	**0.032**

A higher score for functional scales means better functioning. (PF2—physical functioning; RF2—role functioning; EF—emotional functioning; CF—cognitive functioning; SF—social functioning; QL2—global health status/QoL; QLQ-C30 SS—summary score).

**Table 3:** The analysis of variables in symptom scales in EORTC QLQ-C30.

Variables	FA Mean (SD)	NV Mean (SD)	PA Mean (SD)	DY Mean (SD)	SL Mean (SD)	AP Mean (SD)	CO Mean (SD)	DI Mean (SD)	FI Mean (SD)
Age									
<50 years (N = 67)	24.54 (21.75)	3.23 (8.32)	17.66 (21.68)	6.46 (16.65)	26.86 (29.72)	4.47 (15.23)	10.44 (21.87)	2.48 (10.56)	16.41 (27.44)
≥50 years (N = 540)	28.72 (24.63)	5.64 (15.07)	24.59 (26.53)	11.72 (23.11)	32.71 (29.69)	11.79 (23.48)	13.14 (22.56)	4.69 (14.45)	19.44 (26.36)
*p*-value	0.280	0.519	0.064	0.076	0.112	**0.008**	0.208	0.209	0.203
Menopausal status									
Premenopausal (N = 62)	24.73 (22.26)	4.03 (10.31)	17.47 (21.85)	5.91 (15.41)	27.95 (29.68)	5.37 (16.18)	10.21 (21.41)	2.68 (10.96)	15.05(26.08)
Menopausal (N = 545)	28.66 (24.56)	5.53 (14.90)	24.55 (26.48)	11.74 (23.14)	32.53 (29.72)	11.62 (23.38)	13.15 (22.60)	4.64 (14.391)	19.57 (26.51)
*p*-value	0.300	0.767	0.061	0.068	0.233	**0.034**	0.218	0.284	0.124
Cancer staging									
Stage A = 0, I, II (N = 338)	24.91 (23.11)	5.27 (14.09)	21.54 (24.93)	9.76 (21.94)	30.27 (29.28)	9.36 (21.35)	12.03 (22.82)	3.84 (13.15)	17.35 (25.44)
Stage B = III, IV (N = 269)	32.46 (25.24)	5.51 (15.01)	26.70 (27.32)	12.88 (23.197)	34.32 (30.18)	13.01 (24.43)	13.87 (22.05)	5.20 (15.16)	21.31 (27.62)
*p*-value	**<0.001**	0.864	**0.020**	**0.027**	0.109	**0.038**	0.106	0.181	0.073
Type of surgery									
Mastectomy (N = 411)	27.98 (23.98)	4.58 (12.80)	23.56 (26.12)	11.51 (22.86)	31.95 (29.37)	10.38 (22.09)	13.05 (22.56)	4.78 (14.58)	21.00 (27.03)
Conservative breast surgery (N = 127)	23.97 (22.46)	5.11 (14.47)	20.21 (22.28)	8.13 (19.57)	29.39 (29.87)	8.66 (20.66)	11.28 (21.50)	2.10 (9.151)	13.64 (24.61)
*p*-value	0.105	0.944	0.402	0.104	0.349	0.406	0.405	0.058	**0.002**

A higher score for symptom scales means worse functioning. (FA—fatigue; NV—nausea and vomiting; PA—pain; DY—dyspnea; SL—insomnia; AP—appetite loss; CO—constipation; DI—diarrhea; FI—financial difficulties).

**Table 4:** The analysis of variables in functional scales in EORTC QLQ-BR45.

Variables	Body Image Mean (SD)	Future Perspective Mean (SD)	Sexual Functioning Mean (SD)	Sexual Enjoyment Mean (SD)	Breast Satisfaction Mean (SD)
Age					
<50 years (N = 67)	75.62 (29.49)	55.22 (33.61)	71.14 (26.20)	48.64 (28.96)	11.29 (18.16)
≥50 years (N = 540)	84.04 (24.05)	56.11 (34.30)	95.40 (14.64)	40.52 (21.41)	17.43 (23.63)
*p*-value	**0.022**	0.768	**<0.001**	0.155	0.060
Menopausal status					
Premenopausal (N= 62)	76.21 (28.66)	55.37 (34.64)	73.11 (26.54)	47.91 (29.25)	11.01 (17.77)
Menopausal (N= 545)	83.89 (24.25)	56.08 (34.18)	94.95 (15.24)	41.66 (22.24)	17.42 (23.63)
*p*-value	**0.028**	0.842	**<0.001**	0.263	0.057
Cancer staging					
Stage A = 0, I, II (N = 338)	83.70 (25.01)	58.87 (33.50)	92.55 (18.23)	41.49 (24.08)	16.61 (23.87)
Stage B = III, IV (N = 269)	82.37 (24.61)	52.41 (34.78)	92.93 (17.68)	47.00 (26.17)	16.98 (22.06)
*p*-value	0.372	**0.020**	0.861	0.311	0.492
Type of surgery					
Mastectomy (N = 411)	80.47 (26.53)	55.96 (34.27)	93.59 (16.77)	46.29 (27.02)	17.56 (23.49)
Conservative breast surgery (N = 127)	89.56 (18.78)	59.84 (33.15)	89.23 (21.57)	37.33 (20.00)	13.88 (21.98)
*p*-value	**0.001**	0.271	**0.017**	0.198	0.072

A higher score for functional scales means better functioning.

**Table 5:** The analysis of variables in symptom scales in EORTC QLQ-BR45.

Variables	SYS Mean (SD)	HU Mean (SD)	ARM Mean (SD)	BR Mean (SD)	ET Mean (SD)	SM Mean (SD)	ES Mean (SD)
Age							
<50 years (N = 67)	19.82 (14.22)	20.83 (25.65)	21.72 (23.08)	15.17 (12.96)	20.19 (15.18)	10.28 (10.64)	21.02 (25.92)
≥50 years (N = 540)	18.51 (16.10)	30.09 (32.69)	18.62 (20.19)	10.58 (13.17)	21.19 (15.52)	15.01 (13.86)	7.79 (16.63)
*p*-value	0.235	0.243	0.371	**<0.001**	0.556	**0.007**	**<0.001**
Menopausal status							
Premenopausal (N = 62)	20.20 (13.86)	25.00 (29.89)	21.14 (22.91)	14.65 (13.04)	20.26 (14.10)	9.94 (10.06)	20.16 (26.29)
Menopausal (N = 545)	18.48 (16.11)	29.52 (32.33)	18.71 (20.25)	10.68 (13.18)	21.18 (15.63)	15.00 (13.885)	8.01 (16.79)
*p*-value	0.145	0.565	0.523	**0.005**	0.798	**0.007**	**<0.001**
Cancer staging							
Stage A = 0, I, II (N = 338)	17.78 (16.12)	30.03 (32.83)	17.52 (20.04)	10.45 (12.85)	20.04 (15.31)	13.28 (12.82)	9.36 (18.51)
Stage B = III, IV (N = 269)	19.75 (15.57)	27.89 (31.27)	20.77 (21.03)	11.89 (13.63)	22.39 (15.61)	16.00 (14.44)	9.10 (18.16)
*p*-value	**0.030**	0.701	**0.028**	0.148	**0.046**	**0.015**	0.765
Type of surgery							
Mastectomy (N = 411)	18.41 (15.86)	28.43 (31.56)	20.33 (21.31)	11.13 (13.33)	21.21 (15.57)	14.93 (13.27)	9.16 (19.22)
Conservative breast surgery (N = 127)	17.81 (15.84)	31.66 (32.86)	15.04 (18.28)	9.18 (11.91)	11.94 (12.79)	11.94 (12.79)	9.97 (17.22)
*p*-value	0.587	0.588	**0.009**	0.110	0.260	**0.006**	0.212

A higher score for symptom scales means worse functioning. (SYS—systemic therapy side effects; HU—upset by hair loss; ARM—arm symptoms; BR—breast symptoms; ET—endocrine therapy symptoms; SM—skin mucosis symptoms; ES—endocrine sexual symptoms).

**Table 6:** The assessment of QoL in patients diagnosed with breast cancer and heart failure, kidney failure, and neurologic dysfunction by using EORTC QLQ-C30 and EORTC QLQ-BR45.

Items			Heart Failure				Kidney Failure			Neurologic Dysfunction		
		N	Mean (SD)	*p*-Value		N	Mean (SD)	*p*-Value		N	Mean (SD)	*p*-Value
QLQ-C30												
Functional scales												
Physical functioning	HF−	356	77.80 (17.50)	**<0.001**	KF−	500	74.62 (19.52)	**0.018**	N−	502	73.49 (20.75)	0.795
	HF+	251	67.30 (22.96)		KF+	107	68.03 (24.34)		N+	105	73.33 (19.87)	
Role functioning	HF−	356	77.80 (17.50)	0.086	KF−	500	75.10 (27.34)	0.415	N−	502	75.06 (27.67)	0.298
	HF+	251	71.91 (29.52)		KF+	107	72.27 (29.31)		N+	105	72.38 (27.81)	
Emotional functioning	HF−	356	76.42 (22.70)	0.098	KF−	500	77.16 (22.10)	0.074	N−	502	78.25 (22.099)	0.061
	HF+	251	79.58 (21.16)		KF+	107	80.37 (22.08)		N+	105	75.23 (21.13)	
Cognitive functioning	HF−	356	82.72 (21.49)	0.318	KF−	500	82.56 (21.23)	0.268	N−	502	82.30 (21.44)	0.567
	HF+	251	81.80 (20.13)		KF+	107	81.30 (19.52)		N+	105	82.54 (18.40)	
Social functioning	HF−	356	89.37 (21.86)	0.615	KF−	500	88.83 (2.09)	0.701	N−	502	88.87 (21.98)	0.459
	HF+	251	88.84 (21.74)		KF+	107	90.65 (20.24)		N+	105	90.47 (20.78)	
Symptom scales												
Fatigue	HF−	356	26.96 (24.06)	0.112	KF−	500	22.75 (23.75)	0.487	N−	502	28.02 (24.61)	0.402
	HF+	251	30.10 (24.68)		KF+	107	30.63 (26.95)		N+	105	29.14 (23.11)	
Nausea and vomiting	HF−	356	5.47 (14.65)	0.840	KF−	500	5.20 (14.10)	0.617	N−	502	5.37 (14.51)	0.826
	HF+	251	5.24 (14.30)		KF+	107	6.23 (16.27)		N+	105	5.39 (14.52)	
Pain	HF−	356	22.09 (25.76)	**0.034**	KF−	500	23.56 (25.98)	0.602	N−	502	22.61 (26.23)	**0.002**
	HF+	251	26.29 (26.48)		KF+	107	25.07 (26.83)		N+	105	29.68 (24.89)	
Dyspnea	HF−	356	9.83 (20.32)	0.190	KF−	500	10.60 (21.44)	0.590	N−	502	11.28 (22.40)	0.642
	HF+	251	13.01 (25.27)		KF+	107	13.70 (27.07)		N+	105	10.47 (23.25)	
Insomnia	HF−	356	29.213 (28.22)	**0.010**	KF−	500	31.20 (29.84)	0.091	N−	502	31.93 (30.08)	0.671
	HF+	251	36.12 (31.35)		KF+	107	36.13 (29.00)		N+	105	32.69 (28.11)	
Appetite loss	HF−	356	9.83 (21.84)	0.129	KF−	500	10.40 (21.99)	0.282	N−	502	10.95 (22.81)	0.955
	HF+	251	12.61 (24.13)		KF+	107	13.70 (26.28)		N+	105	11.11 (22.95)	
Constipation	HF−	356	11.61 (21.43)	0.112	KF−	500	12.80 (22.54)	0.792	N−	502	12.94 (22.84)	0.920
	HF+	251	14.60 (23.82)		KF+	107	13.04 (22.31)		N+	105	12.38 (20.80)	
Diarheea	HF−	356	3.83 (13.27)	0.118	KF−	500	4.53 (14.05)	0.497	N−	502	4.51 (14.34)	1.000
	HF+	251	5.31 (15.14)		KF+	107	4.05 (14.26)		N+	105	4.12 (12.82)	
Financial difficulties	HF−	356	19.00 (26.43)	0.898	KF−	500	19.06 (26.29)	0.935	N−	502	19.65 (27.43)	0.748
	HF+	251	19.25 (26.60)		KF+	107	19.31 (27.48)		N+	105	19.65 (27.43)	
Global health status/QoL	HF−	356	73.47 (18.71)	**0.024**	KF−	500	72.78 (18.47)	0.057	N−	502	72.44 (18.68)	0.510
	HF+	251	70.35 (18.46)		KF+	107	69.39 (19.35)		N+	105	70.95 (18.60)	
QLQ-C30 summary score	HF−	356	83.38 (13.80)	**0.010**	KF−	500	82.48 (14.10)	0.481	N−	502	82.33 (14.58)	0.323
	HF+	251	80.47 (15.08)		KF+	107	80.77 (15.76)		N+	105	81.43 (13.55)	
EORTC QLQ-BR45												
functional scales												
Body image	HF−	356	81.22 (26.43)	0.053	KF−	500	82.00 (25.46)	**0.007**	N−	502	82.55 (25.42)	0.309
	HF+	251	85.79 (22.12)		KF+	107	88.31 (20.91)		N+	105	85.79 (21.64)	
Sexual functioning	HF−	356	88.15 (21.54)	**<0.001**	KF−	500	91.53 (19.15)	**<0.001**	N−	502	92.72 (18.01)	0.962
	HF+	251	99.20 (7.25)		KF+	107	98.28 (9.13)		N+	105	92.69 (17.89)	
Sexual enjoyment	HF−	85	44.70 (24.96)	0.117	KF−	84	43.65 (25.33)	0.491	N−	72	44.44 (26.24)	0.860
	HF+	3	22.22 (19.24)		KF+	4	50.00 (19.24)		N+	16	41.66 (19.24)	
Future perspective	HF−	356	53.18 (34.29)	**0.015**	KF-	500	55.40 (34.47)	0.329	N−	502	55.77 (33.97)	0.732
	HF+	251	60.02 (33.73)		KF+	107	58.87 (32.88)		N+	105	57.11 (35.42)	
Breast satisfaction	HF−	356	15.23 (21.86)	0.078	KF−	500	16.55 (23.63)	0.254	N−	445	16.81 (23.41)	0.900
	HF+	251	19.08 (24.87)		KF+	107	17.81 (20.61)		N+	94	16.48 (22.06)	
Symptoms scales												
Systemic therapy side effects	HF−	356	18.70 (16.69)	0.539	KF−	500	18.93 (16.08)	0.347	N−	502	18.69 (16.19)	0.688
	HF+	251	18.59 (14.72)		KF+	107	17.35 (15.00)		N+	105	18.45 (14.49)	
Breast symptoms	HF−	356	12.36 (13.50)	**<0.001**	KF−	500	11.43 (13.74)	0.517	N−	502	11.33 (13.52)	0.528
	HF+	251	9.29 (12.60)		KF+	107	9.50 (10.33)		N+	105	9.92 (11.61)	
Arm symptoms	HF−	356	19.85 (21.14)	0.240	KF−	500	19.37 (20.75)	0.278	N−	502	19.21 (20.93)	0.850
	HF+	251	17.70 (19.60)		KF+	107	17.03 (19.44)		N+	105	17.77 (18.52)	
Upset by hair loss	HF−	114	30.11 (32.57)	0.576	KF−	166	29.51 (32.70)	0.747	N−	171	29.04 (31.84)	0.891
	HF+	85	27.45 (31.36)		KF+	33	26.26 (28.57)		N+	28	28.57 (33.59)	
Endocrine therapy symptoms	HF−	356	20.27 (15.70)	0.054	KF−	500	21.32 (15.65)	0.507	N−	502	20.70 (15.78)	0.063
	HF+	251	22.24 (15.10)		KF+	107	20.00 (14.65)		N+	105	22.92 (13.84)	
Skin mucosis symptoms	HF−	356	13.73 (13.37)	0.065	KF−	500	14.50 (13.70)	0.928	N−	502	14.48 (14.21)	0.230
	HF+	251	15.56 (13.92)		KF+	107	14.43 (13.30)		**N+**	105	14.49 (10.408)	
Endocrine sexual symptoms	HF−	356	11.84 (20.95)	**<0.001**	KF−	500	10.08 (19.09)	**0.009**	N− N+	502	9.04 (18.21)	0.256
	HF+	251	5.57 (12.99)		KF+	107	5.37 (13.74)			105	10.23 (18.99)	

**Table 7:** The assessment of QoL in patients diagnosed with breast cancer and liver failure, respiratory failure, and gastrointestinal dysfunction by using EORTC QLQ-C30 and EORTC QLQ-BR45.

Items		Liver Failure				Respiratory Failure					GI Dysfunction		
		N	Mean (SD)	*p*-Value		N	Mean (SD)	*p*-Value			N	Mean (SD)	*p*-Value
QLQ-C30													
Functional scales													
Physical functioning	LF−	526	73.96 (20.23)	0.248	RF−	515	74.82 (19.72)	**<0.001**	GI−		550	73.45 (20.77)	0.827
	LF+	81	70.20 (22.63)		RF+	92	65.87 (23.59)		GI+		57	73.56 (18.89)	
Role functioning	LF−	526	75.57 (27.12)	0.054	RF−	515	76.37 (26.66)	**<0.001**	GI−		550	74.78 (27.50)	0.698
	LF+	81	68.31 (30.57)		RF+	92	64.67 (31.23)		GI+		57	72.80 (29.65)	
Emotional functioning	LF−	526	78.24 (22.03)	0.105	RF−	515	78.35 (21.74)	0.137	GI−		550	77.87 (22.11)	0.536
	LF+	81	74.38 (22.50)		RF+	92	74.27 (23.91)		GI+		57	76.31 (22.26)	
Cognitive functioning	LF−	526	83.08 (20.59)	**0.018**	RF−	515	83.07 (20.63)	**0.037**	GI−		550	82.18 (21.04)	0.496
	LF+	81	77.57 (22.54)		RF+	92	78.26 (22.20)		GI+		57	83.91 (19.91)	
Social functioning	LF−	526	89.63 (21.32)	0.209	RF−	515	90.64 (20.38)	**<0.001**	GI−		550	89.09 (21.97)	0.883
	LF+	81	86.00 (24.36)		RF+	92	80.79 (26.94)		GI+		57	89.76 (19.85)	
Symptoms scales													
Fatigue	LF−	526	27.60 (23.99)	0.147	RF−	515	26.88 (23.35)	**0.006**	GI−		550	28.50 (24.41)	0.449
	LF+	81	32.51 (26.33)		RF+	92	35.99 (28.21)		GI+		57	25.92 (23.88)	
Nausea and vomiting	LF−	526	5.29 (14.80)	0.086	RF−	515	5.08 (13.89)	0.427	GI−		550	5.36 (14.43)	0.829
	LF+	81	5.96 (12.44)		RF+	92	7.06 (17.51)		GI+		57	5.55 (15.21)	
Pain	LF−	526	23.38 (25.53)	0.542	RF−	515	22.33 (25.07)	**0.003**	GI−		550	23.81 (26.08)	0.997
	LF+	81	26.74 (29.66)		RF+	92	32.24 (30.14)		GI+		57	23.97 (26.72)	
Dyspnea	LF−	526	10.83 (22.22)	0.477	RF−	515	9.83 (21.16)	**0.001**	GI−		550	11.39 (22.83)	0.437
	LF+	81	13.16 (24.54)		RF+	92	18.47 (28.11)		GI+		57	8.77 (19.44)	
Insomnia	LF−	526	31.36 (29.35)	0.168	RF−	515	30.48 (29.04)	**0.003**	GI−		550	32.42 (29.79)	0.382
	LF+	81	36.62 (31.88)		RF+	92	40.94 (32.06)		GI+		57	28.65 (29.16)	
Appetite loss	LF−	526	10.83 (22.79)	0.660	RF−	515	10.03 (22.02)	**0.007**	GI−		550	10.97 (23.00)	0.682
	LF+	81	11.93 (23.15)		RF+	92	16.30 (26.37)		GI+		57	11.11 (21.20)	
Constipation	LF−	526	12.42(22.10)	0.264	RF−	515	11.97 (21.73)	**0.033**	GI−		550	12.35 (21.77)	0.264
	LF+	81	15.63 (24.76)		RF+	92	17.75 (25.89)		GI+		57	17.54 (28.24)	
Diarheea	LF−	526	4.30 (13.58)	0.792	RF−	515	4.33 (13.65)	0.852	GI−		550	4.18 (13.67)	0.153
	LF+	81	5.35 (17.04)		RF+	92	5.07 (16.34)		GI+		57	7.01 (17.52)	
Financial difficulties	LF−	526	18.88 (26.55)	0.459	RF−	515	18.18 (26.04)	**0.035**	GI−		550	19.09 (26.63)	0.752
	LF+	81	20.57 (26.12)		RF+	92	24.27 (28.43)		GI+		57	19.29 (25.15)	
Global health status/QoL	LF−	526	72.33 (18.30)	0.860	RF−	515	73.33 (17.60)	**0.002**	GI−		550	72.22 (18.74)	0.693
	LF+	81	71.19 (20.91)		RF+	92	65.76 (22.79)		GI+		57	71.78 (18.00)	
QLQ-C30 summary score	LF−	526	82.65 (14.22)	**0.045**	RF−	515	83.25 (13.79)	**<0.001**	GI−		550	82.18 (14.50)	0.781
	LF+	81	79.11 (15.26)		RF+	92	76.15 (16.28)		GI+		57	82.14 (13.54)	
EORTC QLQ-BR45													
Functional scales													
Body image	LF−	526	83.39 (17.52)	0.648	RF−	515	83.57 (24.61)	0.320	GI−		550	82.98 (25.11)	0.843
	LF+	81	81.27 (27.27)		RF+	92	80.52 (25.95)		GI+		57	84.35 (21.99)	
Sexual functioning	LF−	526	92.55 (18.22)	0.575	RF−	515	92.00 (18.63)	**0.005**	GI−		550	92.69 (17.95)	0.624
	LF+	81	93.82 (16.33)		RF+	92	96.73 (13.13)		GI+		57	92.98 (18.35)	
Sexual enjoyment	LF−	77	42.85 (24.69)	0.402	RF−	82	44.30 (25.68)	0.628	GI−		80	45.41 (25.01)	0.083
	LF+	11	51.51 (27.34)		RF+	6	38.88 (13.60)		GI+		8	29.16 (21.36)	
Future perspective	LF−	526	56.71 (33.87)	0.223	RF−	515	57.54 (33.79)	**0.011**	GI−		550	56.24 (34.09)	0.600
	LF+	81	51.44 (36.15)		RF+	92	47.46 (35.37)		GI+		57	53.80 (35.49)	
Breast Satisfaction	LF−	469	16.98 (23.48)	0.751	RF−	480	16.52 (23.02)	0.591	GI−		487	16.87 (23.32)	0.780
	LF+	70	15.23 (20.99)		RF+	59	18.64 (24.38)		GI+		52	15.70 (21.74)	
Symptoms scales													
Systemic therapy side effects	LF−	526	18.58 (15.96)	0.582	RF−	515	18.07 (15.50)	0.063	GI+		550	18.61 (15.75)	0.889
	LF+	81	19.10 (15.54)		RF+	92	21.89 (17.67)		GI−		57	19.04 (17.35)	
Breast symptoms	LF−	526	11.15 (13.411)	0.950	RF−	515	10.55 (12.53)	0.062	GI+		550	11.06 (13.00)	0.966
	LF+	81	10.70 (11.94)		RF+	92	14.13 (16.28)		GI−		57	11.40 (15.23)	
Arm symptoms	LF−	526	19.24 (20.66)	0.377	RF−	515	18.87 (20.39)	0.971	GI+		550	19.07 (20.64)	0.783
	LF+	81	17.14 (19.72)		RF+	92	19.44 (21.39)		GI−		57	17.93 (19.55)	
Upset by hair loss	LF−	172	29.07 (32.36)	0.974	RF−	164	28.25 (32.10)	0.421	GI+		179	29.05 (32.58)	0.854
	LF+	27	28.39 (30.24)		RF+	35	32.38 (31.81)		GI−		20	28.33 (27.09)	
Endocrine therapy symptoms	LF−	526	20.84 (15.61)	0.192	RF−	515	20.88 (15.27)	0.506	GI+		550	21.33 (15.38)	0.126
	LF+	81	22.67 (14.55)		RF+	92	22.21 (16.63)		GI−		57	18.71 (16.30)	
Skin mucosis symptoms	LF−	526	14.18 (13.53)	0.131	RF−	515	13.98 (13.00)	0.104	GI+		550	14.23 (13.52)	0.178
	LF+	81	16.46 (14.12)		RF+	92	17.33 (16.46)		GI−		57	16.95 (14.48)	
Endocrine sexual symptoms	LF−	526	77.78 (17.52)	0.300	RF−	515	10.12 (19.23)	**0.008**	GI+		550	9.71 (18.71)	0.064
	LF+	81	9.41 (18.38)		RF+	92	4.34 (11.01)		GI−		57	4.82 (13.54)	

**Table 8:** The comparison of QoL in patients diagnosed with metastatic breast cancer.

Items	M1 Non-Oss			M1 Oss			*p*-Value
	N	Mean	SD	N	Mean	SD	
QLQ-C30							
Functional scales							
Physical functioning	49	70.47	20.95	74	58.28	25.21	**0.006**
Role functioning	49	68.02	28.83	74	58.10	32.43	0.090
Emotional functional	49	75.34	26.84	74	71.62	21.00	0.130
Cognitive functioning	49	81.63	21.03	74	77.47	23.47	0.369
Social functioning	49	86.39	24.45	74	79.95	28.13	0.115
Symptoms scales							
Fatigue	49	32.42	27.76	74	42.34	28.05	0.053
Nausea and vomiting	49	4.76	12.72	74	11.48	22.22	0.071
Pain	49	24.83	27.66	74	41.44	30.62	**0.002**
Dyspnea	49	16.32	26.46	74	16.21	28.26	0.865
Insomnia	49	39.45	32.39	74	41.44	32.55	0.746
Appetite loss	49	12.92	24.35	74	21.62	29.42	0.088
Constipation	49	12.24	21.18	74	20.27	26.92	0.100
Diarrhea	49	3.40	12.25	74	8.10	20.50	0.185
Financial difficulties	49	23.81	28.05	74	20.72	26.28	0.541
Global health status/ QoL	49	70.91	18.79	74	59.12	21.77	**0.004**
QLQ-C30 summary score	49	79.65	16.34	74	72.50	16.93	**0.019**
EORTC QLQ BR-45							
Functional scales							
Body image	49	82.48	26.80	74	86.48	23.47	0.549
Sexual functioning	49	96.59	14.01	74	94.14	16.87	0.287
Sexual enjoyment	3	33.33	0.00	9	44.44	23.57	N
Future perspective	49	48.29	36.68	74	44.59	34.13	0.588
Breast satisfaction	37	13.06	16.72	43	20.15	25.08	0.261
Symptoms scales							
Systemic therapy side effects	49	17.88	16.13	74	22.52	17.41	0.116
Breast symptoms	49	11.73	13.91	74	13.85	16.10	0.390
Arm symptoms	49	19.04	21.27	74	22.52	21.12	0.305
Upset by hair loss	12	41.66	20.71	24	27.77	34.98	0.075
Endocrine therapy symptoms	49	17.95	13.90	74	26.26	16.44	**0.002**
Skin mucosis symptoms	49	13.94	13.32	74	19.52	16.71	0.052
Endocrine sexual symptoms	49	3.57	9.62	74	7.09	15.88	0.372

**Table 9:** Multiple linear regression model with estimates for QLQ-C30.

Variable	Age ≥ 50		Stage B		RF+		HF+		LF+		KF+		N+			
	ꞵ	Sig.	ꞵ	Sig.	ꞵ	Sig.	ꞵ	Sig.	ꞵ	Sig.	ꞵ	Sig.	ꞵ	Sig.	Adj. R^2^	*p* Value
QL2	−0.006	0.889	−0.073	0.075	−0.126	0.002	−0.060	0.155	0.011	0.795	−0.053	0.201	−0.029	0.201	0.024	**0.003**
PF2	−0.084	0.036	−0.122	0.002	−0.107	0.007	−0.206	<0.001	−0.025	0.521	−0.063	0.114	−0.004	0.917	0.097	**<0.001**
RF2	−0.006	0.880	−0.138	<0.001	−0.110	0.008	−0.065	0.122	−0.055	0.172	−0.017	0.669	−0.032	0.424	0.041	**<0.001**
EF	0.023	0.587	−0.056	0.174	−0.054	0.195	0.062	0.148	−0.048	0.240	0.044	0.293	−0.045	0.262	0.009	**0.083**
CF	−0.025	0.556	−0.021	0.613	−0.065	0.121	−0.005	0.904	−0.077	0.064	−0.012	0.779	0.006	0.877	0.003	**0.283**
SF	0.018	0.663	−0.035	0.395	−0.153	<0.001	−0.010	0.813	−0.031	0.446	0.040	0.335	0.035	0.390	0.020	**0.007**
FA	0.044	0.294	0.133	0.001	0.098	0.018	0.037	0.382	0.038	0.354	0.024	0.556	0.018	0.650	0.031	**<0.001**
PA	0.072	0.085	0.046	0.062	0.110	0.008	0.051	0.225	0.016	0.686	−0.0006	0.988	0.100	0.013	0.032	**<0.001**
DY	0.057	0.178	0.047	0.254	0.122	0.004	0.038	0.375	0.009	0.823	0.031	0.457	−0.015	0.709	0.018	**0.014**
SL	0.032	0.449	0.044	0.280	0.102	0.014	0.089	0.035	0.036	0.384	0.036	0.387	0.009	0.826	0.021	**0.006**
AP	0.090	0.033	0.068	0.101	0.081	0.053	0.023	0.596	−0.005	0.899	0.034	0.417	0.003	0.950	0.014	**0.029**
CO	0.024	0.573	0.024	0.563	0.078	0.063	0.054	0.204	0.033	0.417	−0.014	0.731	−0.012	0.770	0.003	**0.277**
DI	0.044	0.303	0.048	0.251	0.003	0.947	0.044	0.301	0.021	0.620	−0.031	0.462	−0.012	0.776	−0.004	**0.694**
FI	0.040	0.350	0.064	0.126	0.071	0.091	−0.012	0.777	0.005	0.895	−0.006	0.885	−0.048	0.239	0.003	**0.283**
SS	−0.057	0.168	−0.102	0.012	−0.142	<0.001	−0.068	0.107	−0.048	0.232	−0.015	0.708	−0.020	0.620	0.044	**<0.001**

QL2—global health status; PF2—physical functioning; RF2—role functioning; EF—emotional functioning; CF—cognitive functioning; SF—social functioning; FA—fatigue; PA—pain; DY— dyspnea; SL—insomnia; AP—appetite loss; CO—constipation; DI—diarrhea; FI—financial difficulties; SS—summary score.

**Table 10:** Multiple linear regression model with estimates for QLQ-BR45.

Variable	Age ≥ 50		Stage B		RF+		HF+		LF+		KF+		N+			
	ꞵ	Sig.	ꞵ	Sig.	ꞵ	Sig.	ꞵ	Sig.	ꞵ	Sig.	ꞵ	Sig.	ꞵ	Sig.	Adj. R^2^	*p* Value
BI	0.081	0.055	−0.015	0.716	−0.049	0.240	0.060	0.159	−0.026	0.530	0.079	0.056	0.057	0.160	**0.016**	**0.018**
FU	−0.025	0.549	−0.076	0.065	−0.096	0.021	0.110	0.010	−0.034	0.403	0.029	0.483	0.022	0.591	**0.020**	**0.008**
SX	0.364	<0.001	0.011	0.757	0.062	0.096	0.192	<0.001	0.006	0.863	0.050	0.176	0.009	0.803	**0.216**	**<0.001**
SE	−0.206	0.073	0.152	0.188	−0.077	0.506	−0.151	0.176	0.084	0.448	0.100	0.367	−0.003	0.978	**0.015**	**0.321**
BS	0.065	0.145	0.011	0.793	0.023	0.601	0.064	0.158	−0.030	0.493	0.002	0.968	−0.005	0.903	**0.001**	**0.479**
SYS	−0.021	0.613	0.047	0.255	0.080	0.057	0.003	0.953	−0.004	0.917	−0.039	0.349	−0.011	0.794	**0.000**	**0.411**
HU	0.123	0.104	−0.045	0.536	0.068	0.367	−0.073	0.337	−0.021	0.777	−0.047	0.527	−0.020	0.782	**0.014**	**0.755**
ARM	−0.029	0.487	0.082	0.048	0.007	0.866	−0.038	0.371	−0.041	0.320	−0.033	0.425	−0.030	0.457	**0.002**	**0.313**
BR	−0.082	0.052	0.036	0.381	0.108	0.009	−0.096	0.023	−0.028	0.493	−0.030	0.464	−0.048	0.235	**0.025**	**0.002**
ET	0.014	0.743	0.071	0.089	0.007	0.874	0.067	0.118	0.032	0.434	−0.049	0.243	0.051	0.207	**0.004**	**0.219**
SM	0.106	0.012	0.089	0.030	0.060	0.149	0.037	0.383	0.039	0.338	−0.031	0.458	−0.002	0.954	**0.019**	**0.010**
ES	−0.189	<0.001	0.005	0.906	−0.098	0.016	−0.101	0.015	−0.003	0.949	−0.046	0.251	0.020	0.608	**0.065**	**<0.001**

BI—body image; FU—future perspective; SX—sexual functioning; SE—sexual enjoyment; BS—breast satisfaction; SYS—systemic therapy side effects; HU—upset by hair loss; ARM—arm symptoms; BR—breast symptoms; ET—endocrine therapy symptoms; SM—skin mucosis symptoms; ES—endocrine sexual symptoms.

## Data Availability

Data are only available upon request due to ethical restrictions. The data presented in this study are available upon request from the first author, the corresponding author, and the Coltea Clinical Hospital (secretariat@coltea.ro). The data are not publicly available due to the policy of Coltea Clinical Hospital to have the approval of the Ethics Committee for each new research study.
